# Assessing Habitat Suitability and Exposure of the Endemic *Vasconcellea candicans* Under Climate Change and Urban Growth in Lima, Peru

**DOI:** 10.1002/ece3.74437

**Published:** 2026-10-01

**Authors:** Rebeca M. Cruz‐Zegarra, Jhade R. Gutierrez‐Gutierrez, Abner S. Rivera‐Fernandez, Alexander Cotrina‐Sanchez, Samuel Astete

**Affiliations:** ^1^ Carrera de Ingeniería Ambiental, Facultad de Ingeniería Universidad Peruana de Ciencias Aplicadas Lima Peru; ^2^ Instituto de Investigación Para el Desarrollo Sustentable de Ceja de Selva (INDES‐CES) Universidad Nacional Toribio Rodríguez de Mendoza de Amazonas Chachapoyas Peru; ^3^ Faculty of Agricultural, Environmental and Food Sciences Free University of Bozen/Bolzano Bozen/Bolzano Italy

**Keywords:** biomod2, conservation planning, fog oasis, machine learning, SDM, urban encroachment

## Abstract

Fog oases (lomas) are vegetation islands that support unique biodiversity in the coastal deserts of South America by capturing moisture from seasonal fog. Despite their ecological importance, they are among the region's most threatened ecosystems, particularly around Lima, where informal urbanisation and climate change drive their degradation. However, endemic plants inhabiting these ecosystems remain understudied under combined climatic and anthropogenic pressure. Here, ensemble species distribution modelling was integrated with urban growth projections to jointly quantify climatic and anthropogenic pressures on habitat suitability for *Vasconcellea candicans,* an endemic keystone of Lima's lomas. Occurrence records and environmental predictors were used to calibrate an ensemble of five algorithms under SSP2‐4.5 and SSP5‐8.5 scenarios. Suitability outputs were intersected with current urban land cover and Land Change Modeller projections for 2050 and 2070. Random Forest showed the highest performance (AUC = 0.968, TSS = 0.773), with Temperature Annual Range (bio07) and silt content as the main predictors. The ensemble identified 2891 km^2^ of current high‐suitability habitat; future projections ranged from a 4% loss to a 56% gain by 2070, identifying 2020–2050 as the most critical management window for conservation intervention before urban consolidation forecloses options. Urban expansion has already affected 36.12 km^2^ of high‐suitability habitat and is projected to reach 126.94 km^2^ by 2070 (+251%), intensifying along the southern peri‐urban front, where protected areas coverage remains limited. Formally designating additional lomas units as fragile ecosystems, expanding protection to unprotected high‐suitability patches, and enforcing urban growth boundaries are required to prevent further lomas encroachment.

## Graphical abstract

This study integrated ensemble species distribution modelling and urban growth projections to evaluate current and future habitat suitability of *V. candicans* in the lomas ecosystems surrounding Lima under climate change and urban expansion scenarios. Results identified 2891 km^2^ of current high‐suitability habitat, with strong scenario‐dependent future changes and increasing urban encroachment concentrated along the southern peri‐urban front. The findings highlight the urgent need to expand protected areas, recognise additional fragile ecosystems, and enforce urban growth boundaries to conserve endemic biodiversity in Lima's coastal fog oases.

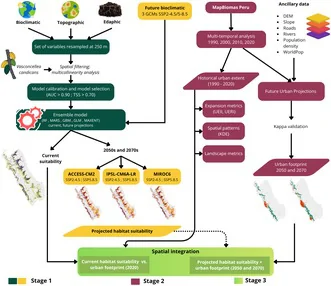

## 1 Introduction

Biodiversity loss is accelerating globally, driven by land‐use change, habitat fragmentation, and climate change, with effects amplified in rapidly urbanising regions (IPBES 2019; IPCC 2023). Coastal desert cities show this pattern clearly, as urban growth competes directly with fog‐dependent vegetation for the same narrow strip of habitable land (Moat et al. 2021). Lima exemplifies the unplanned metropolitan expansion prevalent across South American cities (Ramirez Herrera et al. 2022); severe air pollution from vehicular and industrial sources has made it one of the most polluted cities in Latin America (de Farias et al. 2021; Romero et al. 2020), with direct consequences for its fog oases (known locally as ‘*lomas*’), seasonal ecosystems that represent a natural vegetation in one of the world's largest desert‐built metropolises (Contreras Zuloaga 2023). These ecosystems face the highest anthropogenic stress of any comparable system in Peru (Moat et al. 2021), as informal urbanisation and land‐trafficking drive habitat loss and the physical, chemical, and biological degradation of the soils that sustain them (Camel et al. 2024; Flood Chávez and Niewiadomski 2022).

Fog oases (*lomas*) are seasonal plant communities sustained by dense winter fog banks below 1000 m a.s.l. along the Pacific desert coast of Peru and northern Chile, generated by the interaction of the cold Humboldt Current with the stable subtropical anticyclone (Haas and Dillon 2003; Manrique et al. 2014). This same ocean–atmosphere system also drives the ecosystem's main source of year‐to‐year change: strong El Niño‐Southern Oscillation (ENSO) events replace the lomas' usual fog‐driven seasonality with heavy rainfall, which increases vegetation cover but can lower overall species richness, as a few fast‐growing species dominate the community; normal seasonal patterns return only 1 to 2 years after the event (Tovar et al. 2018). Overall, Peru hosts 67 known *lomas* covering up to 70,000 ha at peak fog incidence, with the highest density concentrated around Lima (Abanto et al. 2021).

In addition to this climate variability, the lomas also face growing human pressure, including informal urbanisation, demographic growth, resource overexploitation, invasive species, and recurrent fires (Aponte et al. 2020), collectively driving declines in biodiversity and vegetation cover (Alonso Quispe and Jesús Solórzano 2021; Pollack et al. 2020). Rising baseline temperatures and shifting precipitation further reduce key ecosystem services, including water regulation, carbon sequestration, and the conservation of forage, medicinal, and crop germplasm (Beresford‐Jones et al. 2015; Dillon and Diebel 2011). Some of these *lomas* have also been officially recognised as fragile ecosystems by the Servicio Nacional Forestal y de Fauna Silvestre (SERFOR), highlighting their vulnerability and conservation urgency (SERFOR 2026). Recognising this importance, Peru's Ministry of the Environment designated five Lima *lomas* as a Regional Conservation Area (MINAM 2020), prioritising climate regulation, water availability, soil stability, carbon sequestration, and the containment of informal urban expansion (Contreras Zuloaga 2023; Guerrero‐Palomino et al. 2022).

*Vasconcellea candicans* (A. Grey) A. DC. (*syn. Carica candicans* A. Grey; locally ‘mito’) is a Critically Endangered endemic species (MIDAGRI 2006) and an ecological keystone of Lima's *lomas*, acting as an umbrella species whose conservation protects the broader community of organisms dependent on its habitat (Isasi‐Catalá 2011; Seoane Byrne and Jeri Kukulis 2018). As an umbrella species, protecting *V. candicans* habitat also protects the other plants and animals that share the same fog‐fed microhabitat, making it a practical focus for conservation across the wider lomas community (Caro 2010). Its capacity for atmospheric water interception makes it a reliable bioindicator of ecosystem health (Eusebio and Mendoza 2006), while its fruit pulp a source of natural antioxidants gives it additional socioeconomic relevance for the food industry (Gutiérrez‐Román et al. 2023). Therefore, understanding the biogeographic distribution and habitat requirements of this species is a conservation priority in a landscape where suitable areas are increasingly restricted and fragmented (Flood Chávez and Niewiadomski 2022; Nieuwland and Mamani 2017).

Species distribution models (SDMs) link occurrence records with environmental and topographic variables to predict species' geographic ranges under current and future conditions (Elith and Leathwick 2009; Miller 2010) and are particularly valuable for endemic taxa with restricted ranges and limited occurrence data (Qazi et al. 2022; Raes et al. 2009). Single‐algorithm approaches such as MaxEnt (Phillips et al. 2006) remain widely used with presence‐only data (Elith et al. 2011; Hernandez et al. 2006). Ensemble SDMs, which combine predictions from algorithms of contrasting statistical structure (GLM, GAM, Random Forest, BRT, MaxEnt) reduce bias and uncertainty inherent in any single approach and produce more robust projections across climate scenarios (Araújo and New 2007; Grenouillet et al. 2011; Hao et al. 2019). Several R packages implement this framework, including biomod2 (Thuiller et al. 2009), SSDM (Schmitt et al. 2017), and sdm (Naimi and Araújo 2016).

Despite growing SDM applications in Peru, ensemble modelling approaches have been focused primarily on crops (e.g., cacao; Ceccarelli et al. 2021; Cotrina‐Sanchez et al. 2026) and fauna (e.g., spectacled bear; Falconi et al. 2023) and rarely been applied to endemic plants under direct urban pressure. Moreover, spatially explicit urban expansion analyses in mid‐sized Peruvian cities (Barboza et al. 2025, 2026) have not been integrated with habitat suitability modelling to jointly quantify climatic and anthropogenic threats on threatened flora. This gap is especially acute in Lima, where urban encroachment on ‘*lomas*’ represents one of the most pressing and spatially quantifiable threats for species such as *V. candicans* . To address this gap, this study combines ensemble SDMs with multi‐temporal urban expansion analysis and land‐use projections to: (i) model the potential distribution of *V. candicans* under current and projected climate change scenarios (2050 and 2070); (ii) characterise historical urban expansion (1990–2020) and project future urban growth to equivalent time horizons; and (iii) spatially integrate both outputs to identify high‐suitability habitat at risk of urban encroachment under current and future conditions and assess its overlap with existing protected areas in Lima.

## 2 Materials and Methods

### 2.1 Study Area

The study area comprised the Department of Lima, Peru extending from 10°16′ 18″ S to 13°19′ 16″ S and 75°30′ 18″ W to 77°53′ 02″ W, covering an area of 35,892.49 km^2^. The department has an arid to semi‐warm climate, with mild temperatures regulated by the Humboldt Current and rainfall restricted to the highlands. Its most ecologically distinctive features are its *lomas*—seasonal plant communities on coastal hillsides below 1000 m a.s.l. (e.g., Lomas de Payet; Figure 1b), sustained by winter fog banks generated by the interaction of the Humboldt Current with coastal orography (Gamboa Moquillaza 2019; Haas and Dillon 2003), with fog concentrated between May and October and mean monthly temperatures spanning ~13.50°C–22.50°C (Manrique et al. 2014; Tovar et al. 2018). Lima harbours the highest *lomas* density in Peru, five of which—Ancón, Carabayllo 1, Carabayllo 2, Amancaes, and Villa María have been designated as the RCA ‘Sistema de Lomas de Lima’ (MINAM 2020) and classified as fragile ecosystems by SERFOR. These lomas are increasingly bordered by informal peri‐urban settlement linked to Lima's rapid population growth (Flores and Van Meerbeek 2024), part of the historical urban expansion pattern quantified in Sections 2.4.1 and 3.4 (Camel et al. 2024; Flood Chávez and Niewiadomski 2022).

**FIGURE 1:**
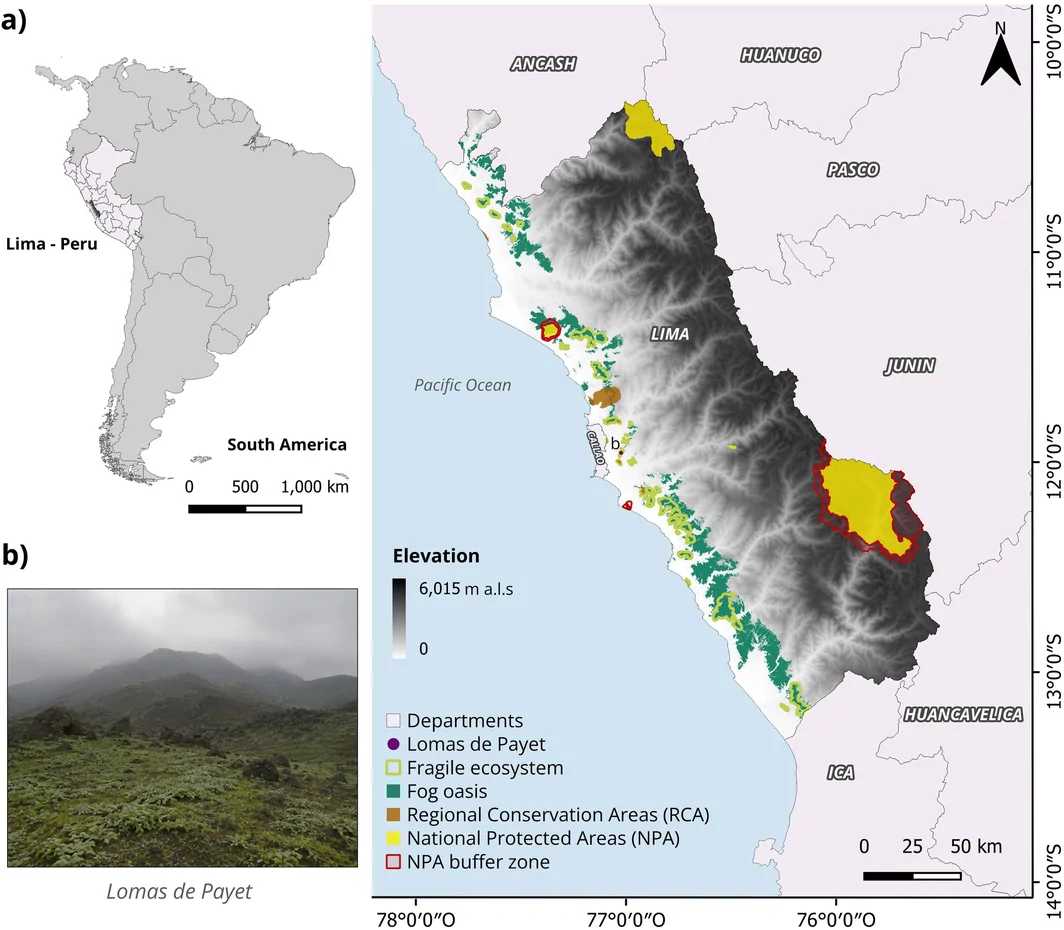
Location of the study area in the department of Lima, Peru, showing the spatial distribution of fog oases, fragile ecosystems, Regional Conservation Areas (RCA), National Protected Areas (NPA), and their buffer zones. (b) Landscape view of ‘Lomas de Payet’ as a representative example of Lima's coastal fog oasis ecosystems. Photo credit: Jhade R. Gutierrez‐Gutierrez.

### 2.2 Methodological Framework

The study comprised three stages (Figure 2): (i) ensemble SDM of *V. candicans* under baseline and projected climate scenarios for 2050 and 2070; (ii) multi‐temporal analysis of historical land cover change (1990–2020) and future urban expansion projections to equivalent time horizons; and (iii) spatial integration of both outputs to identify high‐suitability habitat at risk of urban encroachment and assess its overlap with the existing protected area framework in Lima. This integrated design was adopted because assessing climatic and anthropogenic pressures in isolation tends to over‐ or under‐estimate a species' true conservation status (Nzei et al. 2022; Ramachandran et al. 2020). Stages 1 and 2 generate current and projected suitability surfaces and urban footprints, respectively, for the same time horizons (Figure 2, dark green and dark red blocks), which converge in Stage 3 for spatial overlay (arrows into the light green block). SSP2‐4.5 and SSP5‐8.5 were selected to bracket moderate‐ and high‐emission trajectories rather than sample the full SSP suite (O'Neill et al. 2016; Navarro‐Guzmán et al. 2020), and the 2050s/2070s horizons were kept within the timeframe supported by the 30‐year historical land‐cover baseline (1990–2020) underlying Stage 2, ensuring temporal coherence with Stage 3. Uncertainty is addressed within each upstream stage: via multi‐algorithm ensembling and the six‐GCM × SSP envelope in Stage 1 (Section 2.5; Araújo and New 2007; Goberville et al. 2015), and via Kappa validation in Stage 2 (Section 2.4.2), rather than formally propagated through Stage 3, a limitation acknowledged in Section 4.5.

**FIGURE 2:**
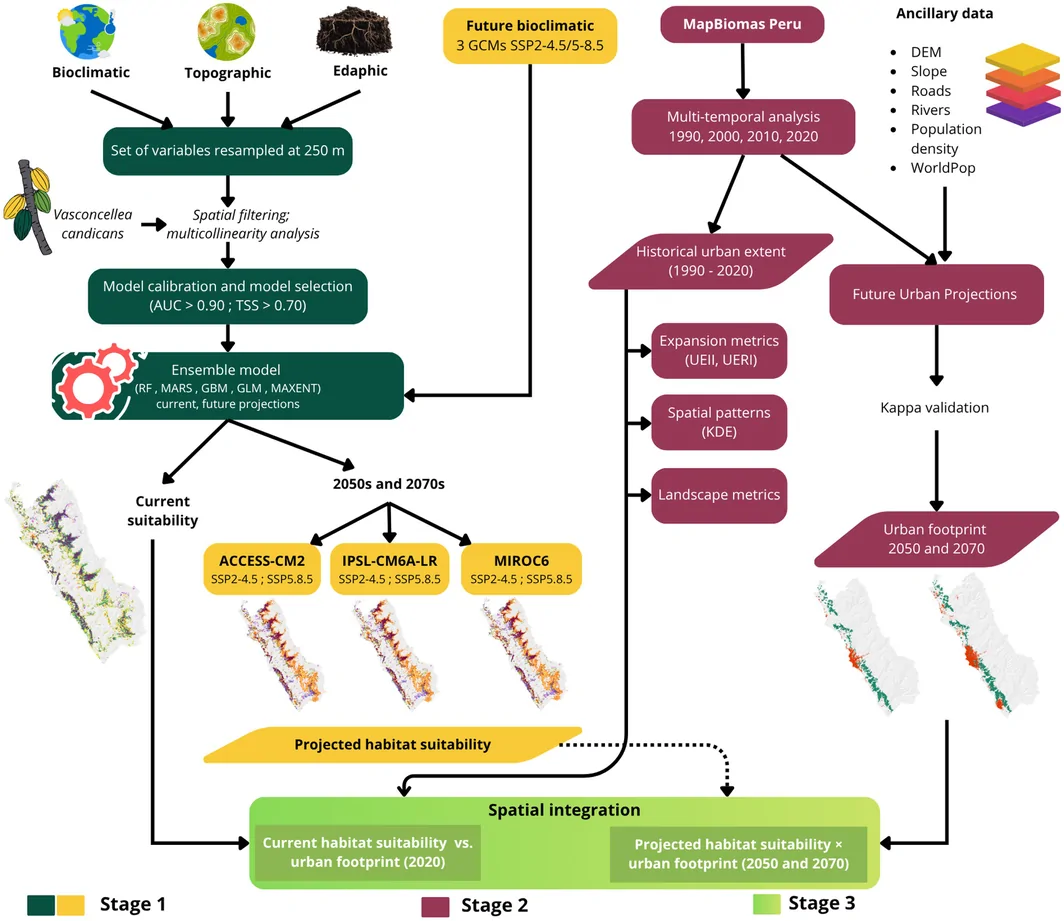
Methodological framework integrating ensemble species distribution modelling and urban expansion analysis to assess habitat loss risk for *Vasconcellea candicans* in Lima, Peru. Stage 1 (dark green): Ensemble SDM calibrated on bioclimatic, topographic, and edaphic variables under three GCMs and two emission scenarios (SSP2‐4.5; SSP5‐8.5) for 2050 and 2070. Stage 2 (dark red): Multi‐temporal land cover analysis (1990–2020) and Land Change Modeller projections of future urban footprint to equivalent time horizons. Stage 3 (light green): Spatial integration of habitat suitability surfaces and urban footprints under current and future conditions.

### 2.3 Ensemble Modelling

#### 2.3.1 Species Occurrence

Occurrence records of *V. candicans* in the department of Lima were downloaded from GBIF (GBIF.org, 12 February 2026; https://doi.org/10.15468/dl.g9hkbs). Records were derived from field observations, herbarium/museum collections, and citizen‐science databases such as iNaturalist (https://www.inaturalist.org), as detailed in Tables S1–S2. Records spanned 1964–2025, with 86.10% collected in 2020–2025 and 93.10% derived from citizen‐science observations on iNaturalist, the remainder from herbarium and DNA‐barcoding voucher specimens (Table S1); because citizen‐science records dominate, the dataset is likely spatially biased toward lomas units more accessible to observers, potentially underrepresenting remote fragments (Boakes et al. 2010). These records were primarily collected between May and October (65.30%), the period of greatest vegetative visibility. Records with coordinate errors, duplicates, or georeferencing inconsistencies were removed, and metadata were cross‐referenced against primary sources available through GBIF Peru (Arenas‐Castro et al. 2022), specifically checking coordinates and taxonomic identification against herbarium specimens from CUVC, MNHN‐Paris, and MO/Field Museum, and against prior floristic records of the species in Lima (Eusebio and Mendoza 2006; Gutiérrez‐Román et al. 2023). To reduce spatial autocorrelation and sampling bias, records were spatially thinned in QGIS v3.40 (QGIS 2026) to one presence point per 250 m grid cell consistent with the resolution of predictor layers (Boria et al. 2014; Veloz 2009). This yielded 101 validated presence records for modelling, and acceptable amount for presence‐background modelling of range‐restricted taxa, as considered in previous studies (Qazi et al. 2022; Raes et al. 2009), though the limited sample size may still underrepresent the species' full environmental niche and inflate variance across model partitions, an effect mitigated in part by evaluating each algorithm over 10 random calibration‐validation partitions (Section 2.3.3). Quality control beyond coordinate‐error and duplicate filtering consisted of spatial thinning; no explicit threshold on reported coordinate uncertainty was applied, though this is partly offset by the spatial thinning procedure itself, which aggregates nearby low‐precision points into shared 250 m grid cells, and by the 10‐partition ensemble approach, which reduces sensitivity to any single record. Phenological stage (flowering/fruiting) was not recorded for any occurrence.

#### 2.3.2 Environmental Variables and Multicollinearity Analysis

A total of 31 environmental variables were initially considered, 19 bioclimatic, 3 topographic, and 9 edaphic (Table S3). Bioclimatic variables representing current (1970–2000) and future conditions (2050s and 2070s) were obtained from WorldClim 2.1 at 30 arc‐seconds (~1 km) (Fick and Hijmans 2017). Future climate projections were obtained from three General Circulation Models (GCMs): ACCESS‐CM2 (Bi et al. 2020), the Institut Pierre‐Simon Laplace Climate Model version 6A low resolution (IPSL‐CM6A‐LR; Boucher et al. 2020), and the Model for Interdisciplinary Research on Climate version 6 (MIROC6; Tatebe et al. 2019) under two Shared Socioeconomic Pathways: SSP2‐4.5, representing a moderate emissions trajectory, and SSP5‐8.5, representing a high‐forcing scenario (Gidden et al. 2019; O'Neill et al. 2017; Riahi et al. 2017). Multiple GCMs were used to capture structural uncertainty arising from differences in model parameterisation and downscaling approaches (Goberville et al. 2015; Wiens et al. 2009). The three GCMs were selected to represent structurally independent modelling centres CSIRO‐BOM, (Australia), IPSL (France), and MIROC (Japan) each with distinct parameterisations and downscaling schemes, rather than the full CMIP6 ensemble, whose members frequently share code and physics and would add redundancy without proportionally reducing structural uncertainty (Goberville et al. 2015).

Topographic variables (elevation, slope, and aspect) were derived from the Shuttle Radar Topography Mission (SRTM) Digital Elevation Model (DEM) at 90 m resolution (Farr et al. 2007). Nine soil variables were obtained from SoilGrids at 250 m resolution (Hengl et al. 2017). All layers were resampled to 250 m (SoilGrids native resolution) in QGIS v. 3.40 (QGIS 2026) using bilinear interpolation, as commonly applied to continuous variables as input in SDMs (Manzoor et al. 2018; Morgan and Guénard 2019). Bioclimatic layers, rather than SoilGrids, were resampled to this common grain because biophysical predictors of finer native resolution are frequently the most influential drivers of SDM accuracy (Manzoor et al. 2018), following standard practice for combining multi‐source layers of differing native resolution (Deneu et al. 2022). Grid cells with missing values in any retained layer were excluded from calibration and projection. No explicit standardisation (e.g., z‐score scaling) was applied to predictors, as the retained algorithms (Section 2.3.3) are scale‐invariant or perform internal normalisation. Topographic and edaphic layers were held constant across all future projections; this assumption is discussed in Section 4.5.

To reduce multicollinearity among predictors, a Variance Inflation Factor (VIF) analysis was conducted in R using the *usdm* package (Naimi et al. 2014), whereby each variable was regressed against all remaining predictors (Arenas‐Castro et al. 2022; Dormann et al. 2013). Variables with a VIF > 5 were iteratively removed to ensure that a low‐redundancy predictor set was achieved (Marquardt 1970; Zhang et al. 2026). This procedure yielded a final set of 12 variables retained for modelling (Table 1).

**TABLE 1:** Environmental variables retained after multicollinearity filtering (VIF < 5) for ensemble species distribution modelling of *V. candicans* .

Variable	Description	Unit	Symbol	VIF
Bioclimatic	Temperature Annual Range (bio5‐bio6)	°C	bio07	2.785
	Precipitation Seasonality (Coefficient of Variation)	mm	bio15	2.508
	Precipitation of Coldest Quarter	mm	bio19	2.809
Topographic	Cardinal orientation of the slope	°	aspect	1.026
	Slope of the terrain	°	slope	1.528
Edaphic at 0.30 m	Apparent density of the fine earth fraction	cg/cm^3^	bdod	4.421
	Cation exchange capacity	cmol kg^−1^	cec	3.751
	Volumetric fraction of coarse fragments	cm^3^/dm^3^ (vol %)	cfvo	1.364
	Total nitrogen	cg/kg	nitrogen	3.291
	Clay content	%	clay	2.844
	Silt content	%	silt	3.512
	Organic carbon	g kg^−1^	soc	4.793

#### 2.3.3 Model Implementation

Ensemble SDMs were implemented in the biomod2 package (Thuiller et al. 2009). As confirmed absences were unavailable, pseudo‐absences were generated using the Surface Range Envelope (SRE) strategy, which selects background points outside the environmentally suitable space defined by a rectilinear envelope fitted to presence data at a quantile threshold of 0.025 (Leta et al. 2019). SRE was preferred over target‐group or geographic‐distance background methods because, for a range‐restricted, poorly surveyed endemic with no confirmed absences, it constrains pseudo‐absences to environmentally distinct space without requiring an a priori accessible‐area assumption (Leta et al. 2019). Five independent pseudo‐absence sets of 1000 points each were generated to minimise the influence of any single background configuration on model outputs. This configuration follows Barbet‐Massin et al. (2012), who found that five replicates of 1000 pseudo‐absences each were sufficient to stabilise ensemble predictions for presence‐only datasets of comparable size.

Nine algorithms were initially evaluated: Generalised Linear Model (GLM), Generalised Additive Model (GAM), Random Forest (RF), Gradient Boosting Machine (GBM), Multivariate Adaptive Regression Splines (MARS), Flexible Discriminant Analysis (FDA), Classification Tree Analysis (CTA), MaxEnt, and Artificial Neural Network (ANN). No formal hyperparameter tuning (biomod2's tuned strategy) was performed for any algorithm; all algorithms were run using biomod2's bigboss parameter set, a pre‐defined configuration recommended over each algorithm's raw software defaults (Thuiller et al. 2009), consistent with other recent biomod2‐based SDM studies (Kingsbury et al. 2025). Each was calibrated on 80% of records and validated on the remaining 20% over 10 random partitions (Cotrina‐Sanchez et al. 2026; Leta et al. 2019). Model performance was assessed using AUC‐ROC (threshold > 0.9; Anderson et al. 2011) and TSS (threshold > 0.7; Allouche et al. 2006). Variable importance was quantified via permutation tests, measuring the decrease in model performance upon random shuffling of each predictor (Thuiller et al. 2009), though permutation‐based importance is known to overestimate the contribution of correlated predictors (Strobl et al. 2008), a bias already constrained by the prior VIF‐based multicollinearity filtering (Section 2.3.2). ANN failed to converge and was excluded prior to evaluation, a pattern of instability common to neural‐network architectures trained on comparatively small presence‐only datasets (Valavi et al. 2022). Of the remaining eight algorithms, five met both thresholds and were retained for ensemble construction (RF, MARS, GBM, GLM, MaxEnt); GAM, FDA, and CTA were excluded due to insufficient performance in at least one metric.

The ensemble model was built using the BIOMOD_Ensemble Modeling function (TSS‐weighted mean; EMwmean), combining individual algorithm predictions proportionally to their validation TSS scores and yielding a continuous suitability surface rescaled to 0–1 (Thuiller et al. 2009). Projections were extended to 2050 and 2070 for each GCM × SSP combination by replacing bioclimatic layers with their future counterparts; topographic and edaphic layers were held constant over the modelled time horizons (Thuiller et al. 2009). The resulting surface was classified into four suitability categories: unsuitable (< 0.2), low (0.2–0.4), moderate (0.4–0.6), and high (> 0.6) (Cotrina‐Sanchez et al. 2026; Zhang et al. 2019), a threshold also applied in other Andean‐forest SDM studies (Ames‐Martínez et al. 2025) and biologically interpretable given that the ensemble's own AUC/TSS inclusion thresholds already restrict retained algorithms to those reliably discriminating presence from background at this suitability level; only areas above the 0.2 threshold were considered in spatial overlay analyses.

### 2.4 Urban Expansion Analysis and Future Urban Growth Projections

#### 2.4.1 Historical Urban Expansion (1990–2020)

Land cover data were obtained from MapBiomas Peru Collection 3.0 (1985–2024), an annual 30 m Landsat‐derived land use and land cover series classified with random forest in Google Earth Engine following the MapBiomas network protocol (Souza Jr. et al. 2020). Accuracy for the Peruvian collection is assessed through a stratified random design described in its ATBD (MapBiomas Perú 2026), based on two domains (inside and outside the RAISG boundary), 19 aggregated map sheets of approximately 70,000 km^2^ within the RAISG domain and six slope strata per domain, with a minimum of 500 points per domain adjusted by class variability and Bonferroni correction; the design yields a maximum standard error of 5% for aggregated sheets and 2.5% for the remaining territory at 95% confidence. Network‐level validation of the MapBiomas time series reported a mean overall accuracy of 89% (range 73%–95%) across 75,000 stratified random sample locations.

Historical urban growth was characterised using the urban and built‐up class (code 24) from MapBiomas Peru Collection 3 at 30 m spatial resolution (MapBiomas Perú 2026), across three inter‐decadal periods (1990–2000, 2000–2010, 2010–2020). The urban class aggregates residential, commercial, industrial, and transport infrastructure without distinguishing them, consistent with standard practice at coarse mapping scales (Anderson et al. 1976). The process of interest is the transformation of vegetated or bare slopes into compacted surfaces, which suppresses the underlying ecosystem regardless of subsequent use; the class therefore precludes disaggregation by activity type, and dispersed informal settlements may be under‐ or over‐represented depending on pixel‐level spectral mixture. Expansion intensity was quantified using the Urban Expansion Intensity Index (UEII) and the Urban Expansion Rate Index (UERI) across different time periods (Hu et al. 2007; Li et al. 2025). The indices are defined as:

$$\text{UEII}=\left(\left(U_{2}-U_{1}\right)/\left(A_{\text{total}}\times ∆t\right)\right)\times 100$$

$$\text{UERI}=\left(\left(U_{2}-U_{1}\right)/\left(U_{1}\times ∆t\right)\right)\times 100$$

where *U*_1_ and *U*_2_ are urban areas (km^2^) at the beginning and end of each period, *A*_total_ is the total area of the study area, and Δ*t* is the interval in years.

The spatial concentration of new urban pixels was characterised via two‐dimensional Gaussian kernel density estimation (KDE) implemented in R using the *spatstat* package, with the analysis window restricted to the bounding box of expansion pixels; bandwidth was selected via pseudo‐likelihood cross‐validation (Baddeley et al. 2015). Urban landscape morphology was assessed at each time step using four class‐level metrics from the *landscapemetrics* package (Hesselbarth et al. 2019): the Landscape Shape Index (LSI; McGarigal et al. 2018), Fractal Dimension Index (FRAC; Mandelbrot 1983), number of patches (NP), and mean patch size (MPS). The Compactness Index (CI; Deng et al. 2021) was also computed:

$$\mathrm{CI}=\frac{2\surd \mathrm{\pi A}}{P}$$

where *A* is total urban area (m^2^) and *P* is total urban perimeter (m), with CI = 1 indicating a perfectly circular form and CI → 0 an elongated or fragmented one.

Finally, expansion was spatially disaggregated by partitioning the department's latitudinal extend (Section 2.1) into tree equal zones: north, central, and south and computing newly urbanised area per zone and period.

#### 2.4.2 Urban Expansion and Future Urban Projections

Future urban expansion was projected to 2050 and 2070 using MOLUSCE v.4.1.0 (Modules for Land Use Change Evaluation), a QGIS (Sclafani Pujatti et al. 2022), that integrates Markov Chain transition probability matrices with spatially explicit transition potential surfaces generated by Multilayer Perceptron (MLP) neural networks and allocates simulated change through cellular automata (Mas et al. 2018). MapBiomas LULC maps were reclassified into eight categories: forest formation, non‐forest formation, agricultural area, non‐vegetated area, urban area, water body, mining, and fog oasis preserving both the urban/non‐urban distinction and fog oasis (MapBiomas class 70) as an independent category, so the projected urban footprint could be evaluated directly against lomas extent. Transition potentials were calibrated on the 2000–2010 interval, validated against the observed 2020 map, then recalibrated on the 2010–2020 interval, the most recent period and best representative of current expansion, for projection to 2050 and 2070 (Sclafani Pujatti et al. 2022). Ten candidate predictors were screened by Pearson correlation with observed urban transitions (retaining |*r*| > 0.5), a standard step for eliminating multicollinearity in cellular automata land use models (Feng and Tong 2017); aspect, distance to railways, distance to district capitals, and existing urban area were discarded for falling below this threshold. Six spatial predictors were incorporated: elevation and slope (DEM from IGN contour lines; IGN 2026), Euclidean distance to population centres, proximity to the road network (MTC 2026), proximity to the hydrographic network (ANA 2026), and population density (WorldPop 2026). MapBiomas class 24 does not distinguish formal from informal settlement, so the model captures only the aggregate built footprint; informal occupation in Lima typically becomes reliably detectable in optical archives only after initial consolidation, in contrast to SAR‐based monitoring capable of detection within days (Moya et al. 2022), so historical rates, and the projections built on them, likely lag the true onset of occupation. All layers were resampled to 30 m via cubic convolution interpolation in QGIS v3.40, consistent with the MapBiomas baseline resolution. Transition potentials were modelled with a multilayer perceptron trained on 1000 randomly sampled pixels, using a neighbourhood of one pixel (a 3 × 3 window) to incorporate local spatial context into the modelled transitions, a single hidden layer of 10 neurons, a learning rate of 0.001, a momentum of 0.05, and a maximum of 500 iterations. Training converged to a minimum total error of 0.00853 and a training kappa of 0.82. These parameter choices are consistent with standard practice in cellular‐automata‐based LULC modelling (Sabri and Khayyun 2024; Rivera‐Fernandez et al. 2026).

Model accuracy was evaluated on a temporally independent basis: transition potentials calibrated on the observed 2000–2010 interval were used to simulate the 2020 land cover map, which was then compared against the observed MapBiomas 2020 map over five validation iterations. The simulation achieved 90.40% correctness and an overall kappa of 0.864, decomposed into a quantity component (κ_histogram = 0.985) and a location component (κ_location = 0.878), indicating that the model reproduces the magnitude of change almost exactly and its spatial allocation with high fidelity (Pontius Jr. 2000). A multiple‐resolution budget analysis (Pontius et al. 2008) returned agreement of 0.906 at the native 30 m resolution, rising to 0.938 under aggregation, against 0.293 for a null model informed by quantity but not location and 0.071 for an uninformed null; the model therefore exceeds the quantity‐only null by 61.3 percentage points, confirming that its skill derives from spatial allocation rather than from correctly reproducing aggregate totals alone. The validated model was then recalibrated on the 2010–2020 interval and applied to project LULC for 2050 and 2070.

### 2.5 Spatial Overlay Analysis of Suitability and Urban Expansion

To identify high‐suitability habitat for *V. candicans* susceptible to urban encroachment and prioritise conservation targets, the ensemble suitability surface (> 0.2 threshold) was overlaid in QGIS v3.40 with the 2020 urban footprint and LCM‐projected urban extents for 2050 and 2070. Given their differing native resolutions (250 m suitability, 30 m urban footprint), the suitability surface was classified first, then resampled to 30 m by nearest‐neighbour assignment to preserve class boundaries without interpolation (Deneu et al. 2022). The intersection between urban areas and each suitability class was quantified in km^2^, with particular attention to high‐suitability zones (> 0.6) as the most critical conservation targets. To bound plausible future suitability outcomes, two GCMs were selected based on contrasting projections: one maximising and one minimising high‐suitability habitat gains, representing high‐projection and low‐projection bounds, respectively (Araújo and New 2007; Khan et al. 2025). Selected GCMs are identified in Section 3.1. Both SSP2‐4.5 and SSP5‐8.5 were retained as separate analytical units per time horizon, yielding four suitability surfaces for 2050 and 2070. Urban projections do not depend on GCM/SSP, since LCM transitions are driven by topographic, accessibility, and demographic covariates; a single urban footprint per time horizon was thus intersected with all four suitability surfaces. All overlap layers were intersected with RCA, NPA, and fragile ecosystem boundaries (SERFOR 2026; SERNANP 2026) to assess the degree to which formally protected areas encompass high‐suitability habitat at risk of urban encroachment.

## 3 Results

### 3.1 Model Performance and Variables Contribution

Five of the seven evaluated algorithms were retained for ensemble construction based on their superior predictive performance (Figure 3a). RF achieved the highest AUC score (AUC = 0.968 ± 0.019; TSS = 0.773 ± 0.078), followed by MARS (AUC = 0.966 ± 0.024; TSS = 0.826 ± 0.081), GBM (AUC = 0.958 ± 0.020; TSS = 0.774 ± 0.079), GLM (AUC = 0.942 ± 0.025; TSS = 0.747 ± 0.077), and MaxEnt (AUC = 0.905 ± 0.035; TSS = 0.765 ± 0.080). GAM, FDA, and CTA were excluded for failing at least one threshold. Notably, MaxEnt and GLM, despite showing the lowest and second‐lowest AUC/TSS among the five retained algorithms assigned the highest mean importance to the top predictors (Figure 3b), whereas RF, the top‐performing algorithm, assigned the lowest. This reflects a known algorithm‐specific behaviour: tree‐ensemble methods such as RF distribute predictive weight across a larger number of correlated or complementary variables, diluting the apparent contribution of any single predictor, whereas regression‐ and exponential‐based methods (GLM, MaxEnt) concentrate importance in fewer dominant variables (Mi et al. 2017; Zhang et al. 2020). MaxEnt's comparatively lower discriminative performance is consistent with benchmark evidence that presence‐background methods are more sensitive to small sample sizes than tree‐based ensembles (Valavi et al. 2022), relevant given the 101 occurrence records used (Section 2.3.1). Despite differences in algorithmic structure, the five retained models converged on a consistent set of dominant environmental predictors (Figure 3b). Bio07 (Temperature Annual Range) and silt content were the most influential predictors, with mean importance values of 27.2% (max: 39.5%, MaxEnt) and 25.4% (max: 38.3%, GLM), respectively. Slope (mean: 20.2%, max: 35.2% in MaxEnt), total nitrogen (mean: 19.0%, max: 31.3% in GLM), and cation exchange capacity (mean: 18.9%, max: 29.9% in MaxEnt) followed in relative contribution. In contrast, aspect showed the lowest mean contribution across all algorithms (mean: 2.4%, max: 6.5% in MaxEnt). The error bars in Figure 3b (± SD across the 10 calibration‐validation partitions, Section 2.3.3) further show that RF had the narrowest SD for all five predictors, indicating the most consistent (albeit lowest‐magnitude) importance estimates, whereas MaxEnt and GLM combined the highest mean importance with the widest SD, indicating greater sensitivity to partition‐specific presence/pseudo‐absence configurations. Formal confidence intervals were not computed, as permutation importance was assessed as a mean ± SD across partitions rather than through a dedicated bootstrap procedure; the reported SD nonetheless provides a direct measure of estimate stability per algorithm. Potential interactions between the top two predictors (Bio07 and silt content) were not formally tested via interaction terms or bivariate response surfaces and are noted as an avenue for future refinement; ecologically, their covariation is plausible given that fog‐derived moisture jointly governs thermal buffering and pedogenic silt accumulation on lomas hillslopes (Section 4.1).

**FIGURE 3:**
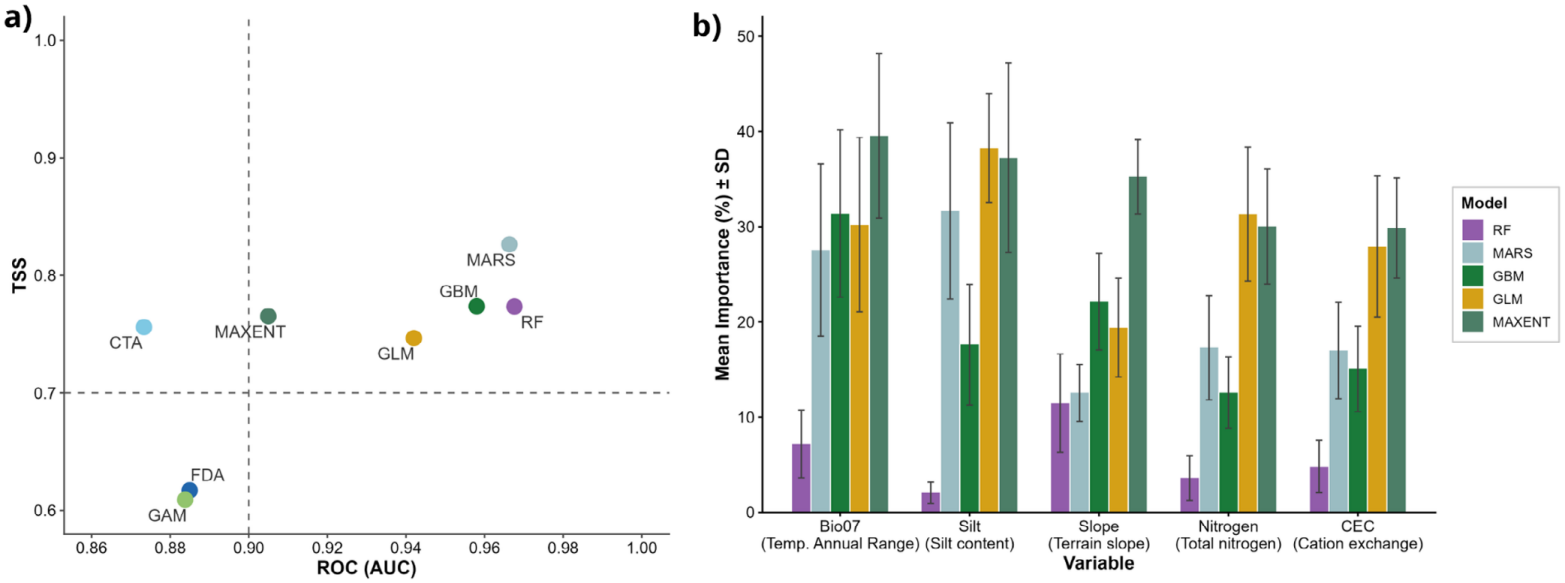
Predictive performance and variable importance of the ensemble algorithms for *Vasconcellea candicans* in Lima, Peru. (a) AUC‐ROC and TSS values for the seven evaluated algorithms; dashed lines indicate minimum inclusion thresholds (AUC > 0.90, TSS > 0.70). (b) Mean importance (%) ± SD of the five most influential predictors across the five retained algorithms.

### 3.2 Current Potential Distribution

The ensemble identified 8500.42 km^2^ of suitable habitat across Lima (24.28% of the department; Figure 4; Table 2), of which high‐suitability areas (> 0.6) accounted for 2891.28 km^2^ (8.26%). Aggregating high‐ and moderate‐suitability classes into a combined ‘Area of Interest’ (AOI), representing areas most relevant for near‐term conservation prioritisation yields 4923.21 km^2^ (14.06% of Lima's territory; Table S4), rising to 34.17% within fog oases, 57.86% within fragile ecosystems, and 20.96% within RCAs, but only 2.40% within NPAs, reinforcing the disproportionate conservation value of RCAs and fragile ecosystems relative to the existing NPA network. Suitable habitat was spatially heterogeneous: fragile ecosystems and fog oases concentrated disproportionate fractions of high‐suitability habitat relative to their extent (46.85% and 25.01%, respectively), with 69.30% and 45.39% of their total areas classified as suitable overall. Within the protected area framework, RCAs showed a higher proportion of high‐suitability habitat (15.15%) than NPAs (1.18%), despite their smaller total extent, underscoring their disproportionate conservation value for the species. Confidence intervals for these area estimates were not computed, as the ensemble surface reported is the TSS‐weighted mean across algorithms (Section 2.3.3) rather than a distribution of per‐partition area values; deriving formal CIs would require retaining and aggregating suitable‐area estimates from each of the 10 calibration‐validation partitions and five algorithms individually, which was beyond the scope of the current workflow and is noted as a direction for future refinement (Section 4.5).

**FIGURE 4:**
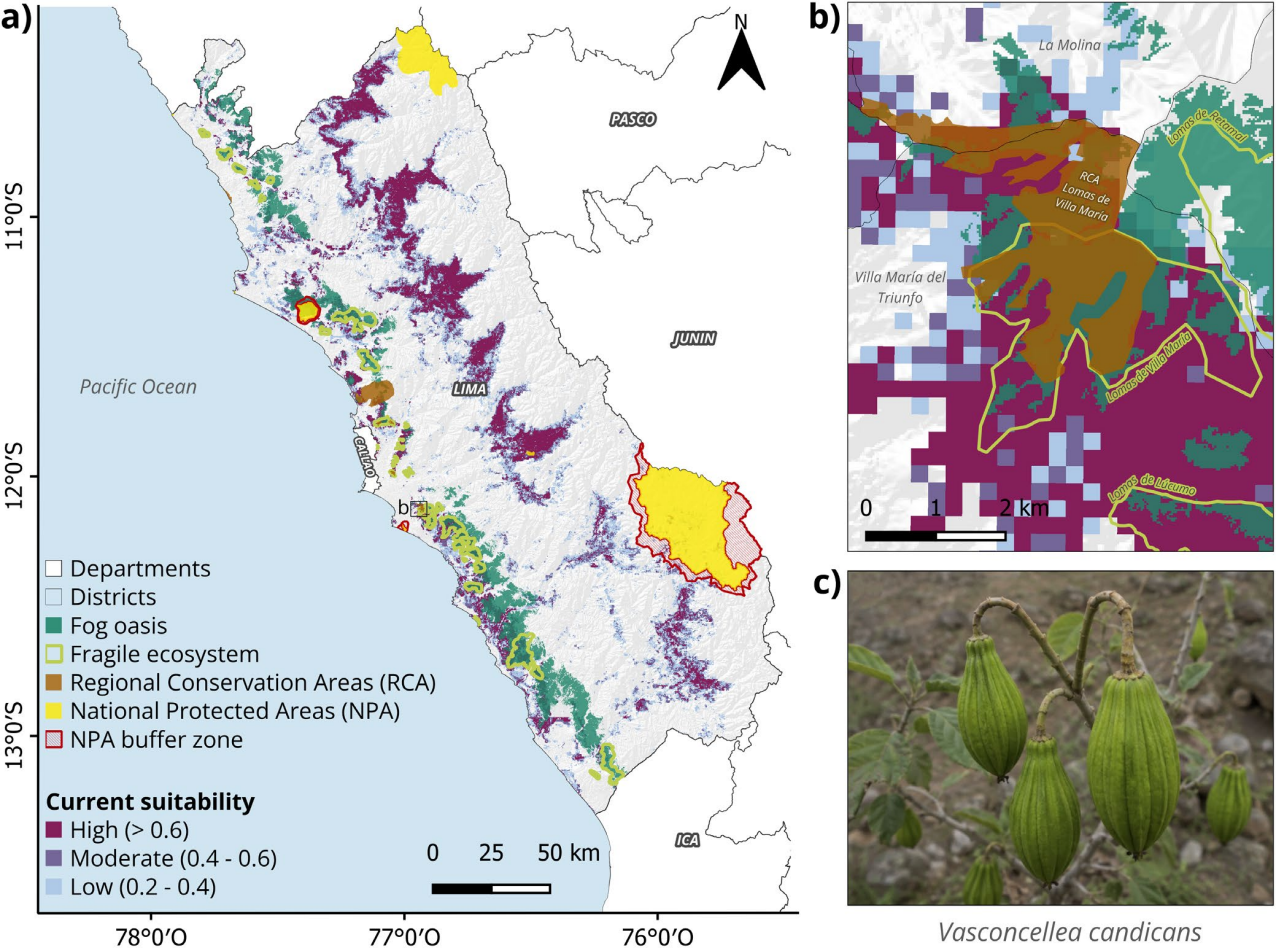
Current potential habitat suitability of *Vasconcellea candicans* in the department of Lima, Peru. (a) Spatial distribution of suitability classes (high: > 0.6; moderate: 0.4–0.6; low: 0.2–0.4) overlaid on fog oases, fragile ecosystems, Regional Conservation Areas (RCAs), and National Protected Areas (NPAs). (b) Detail of high‐suitability areas within the RCA Lomas de Villa María (Villa María del Triunfo district), showing the spatial relationship between habitat suitability and conservation unit boundaries. (c) *Vasconcellea candicans* individuals photographed within the RCA Lomas de Villa María. Photo credit: Jhade R. Gutierrez‐Gutierrez.

**TABLE 2:** Current potential distribution of *Vasconcellea candicans* (km^2^ and percentage) across Lima's territory, fog oases, fragile ecosystems, Regional Conservation Areas (RCA), and National Protected Areas (NPA) by habitat suitability class (High: > 0.6, Moderate: 0.4–0.6, Low: 0.2–0.4). Percentages are calculated relative to the total surface area of each spatial unit.

Area/Ecosystem	High (> 0.6)		Moderate (0.4–0.6)		Low (0.2–0.4)		Total km^2^	Total %
	km^2^	%	km^2^	%	km^2^	%		
Lima's territory	2891.28	8.26	2031.93	5.80	3577.22	10.22	8500.42	24.28
Fog oasis	584.43	25.01	213.93	9.16	262.28	11.23	1060.64	45.39
Fragile ecosystem	275.39	46.85	64.69	11.01	67.21	11.44	407.30	69.30
Regional Conservation Areas (RCA)	21.12	15.15	8.10	5.81	7.73	5.54	36.95	26.50
National Protected Areas (NPA)	21.70	1.18	22.47	1.22	108.91	5.92	153.09	8.32

### 3.3 Projected Potential Distribution

Projected habitat suitability for *V. candicans* varied substantially between bounding scenarios across both time horizons (Table 3; Figure 5). Under the low‐projection scenario (IPSL‐CM6A‐LR SSP2‐4.5), high‐suitability habitat declined progressively from 2818.92 km^2^ (−2.50%) in the 2050s to 2773.22 km^2^ (−4.08%) by the 2070s. The high‐projection scenario (ACCESS‐CM2 SSP5‐8.5) projected a sustained expansion, from 3705.43 km^2^ (+28.16%) to 4520.45 km^2^ (+56.35%) over the same period. The divergence between scenarios intensified toward 2070, with the gap in high‐suitability area widening from 886.51 km^2^ in the 2050s to 1747.23 km^2^ by the 2070s. Complete projections for all six GCM–SSP combinations are provided in Tables S5 and S6 and Figure S1.

**TABLE 3:** Projected habitat suitability of *V. candicans* under bounding climate scenarios for 2050 and 2070 in the department of Lima. Values in parentheses indicate percentage change relative to current conditions (1970–2000). ACCESS‐CM2 and IPSL‐CM6A‐LR represent the high‐projection and low‐projection bounds of the projected distribution envelope, respectively.

Climate scenario		High suitability (> 0.6)		Moderate suitability (0.4–0.6)		Low suitability (0.2–0.4)		Unsuitable (< 0.2)	
Time horizon	GCM—SSP	km^2^ (Δ% vs. current)	% of Lima	km^2^ (Δ% vs. current)	% of Lima	km^2^ (Δ% vs. current)	% of Lima	km^2^ (Δ% vs. current)	% of Lima
Current (1970–2000)		2891.28	8.26	2031.93	5.8	3577.22	10.22	24,952.44	71.27
2050s	SSP2‐4.5 IPSL‐CM6A‐LR	2818.92 (−2.50)	8.05	2010.45 (−1.06)	5.74	3519.33 (−1.62)	10.05	25,100.62 (+0.59)	71.69
	SSP5‐8.5 ACCESS‐CM2	3705.43 (+28.16)	10.58	2348.86 (+15.60)	6.71	3899.15 (+9.00)	11.14	23,495.87 (−5.84)	67.11
2070s	SSP2‐4.5 IPSL‐CM6A‐LR	2773.22 (−4.08)	7.92	1978.97 (−2.61)	5.65	3471.47 (−2.96)	9.92	25,225.65 (+1.09)	72.05
	SSP5‐8.5 ACCESS‐CM2	4520.45 (+56.35)	12.91	2396.34 (+17.93)	6.84	3925.49 (+9.74)	11.21	22,607.03 (−9.40)	64.57

**FIGURE 5:**
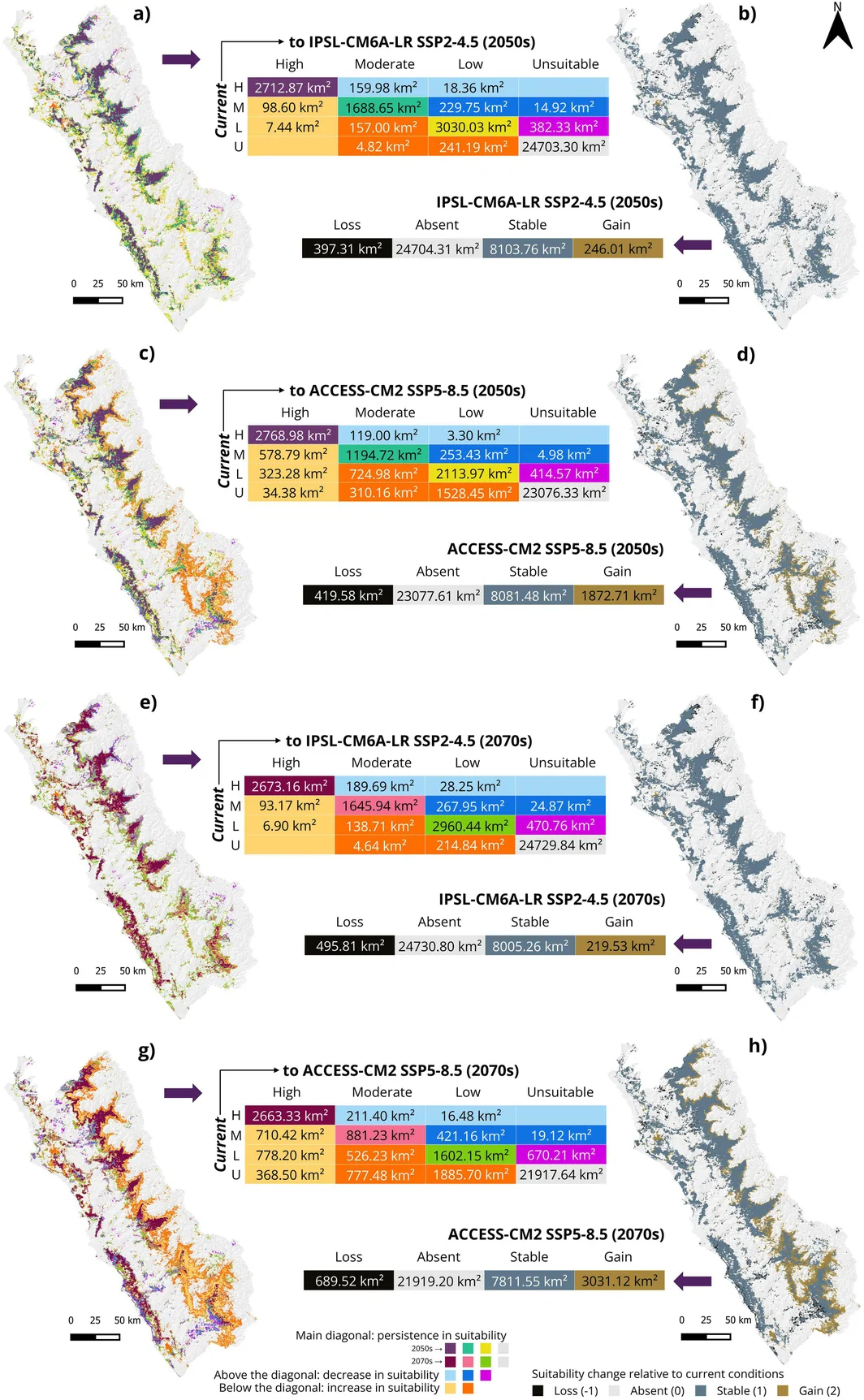
Projected habitat suitability of *Vasconcellea candicans* in the department of Lima under bounding climate scenarios, showing suitability class transitions alongside the corresponding spatial pattern of habitat change. (a, b) IPSL‐CM6A‐LR SSP2‐4.5 (2050s); (c, d) ACCESS‐CM2 SSP5‐8.5 (2050s); (e, f) IPSL‐CM6A‐LR SSP2‐4.5 (2070s); (g, h) ACCESS‐CM2 SSP5‐8.5 (2070s). Suitability classes follow the classification scheme described in Section 2.3.3 (Low: 0.2–0.4; Moderate: 0.4–0.6; High: > 0.6).

### 3.4 Spatial–Temporal Analysis of Urban Growth (1990–2020)

Between 1990 and 2020, Lima's urban footprint expanded from 429.0 to 848.5 km^2^ (net gain: 419.5 km^2^; Table 4). The highest absolute increment occurred in 1990–2000 (+166.1 km^2^; UERI: 3.864%/yr), with subsequent decades showing lower absolute gains (2000–2010: +124.4 km^2^; 2010–2020: +129.0 km^2^) and declining expansion indices (UERI: 2.086 and 1.804%/yr., respectively), indicating a progressive deceleration in relative growth despite continued absolute urban area increase.

**TABLE 4:** Urban area and expansion metrics per inter‐decadal period in the department of Lima, including net area gained, growth rate, Urban Expansion Intensity Index (UEII), and Urban Expansion Rate Index (UERI).

Period	Urban area (km^2^)	New area (km^2^)	Growth (%)	UEII (%/yr)	UERI (%/yr)
1990 (baseline)	429.0	—	—	—	—
1990–2000	594.73	165.75	38.64	0.048	3.864
2000–2010	718.77	124.04	20.89	0.036	2.086
2010–2020	848.41	129.64	18.04	0.037	1.804
1990–2020	848.41	419.43	97.78	0.040	3.259

Urban expansion was concentrated in the central zone throughout the study period, consistently accounting for 84%–93% of new urban area per decade (Table 5), reflecting the dominant role of Lima Metropolitana and Callao. Nevertheless, the southern zone increased its proportional contribution from 2.7% (1990–2000) to 8.8% (2010–2020), driven by periurban expansion in Lurín, Pachacámac, and Villa María del Triunfo (Figure 6a); the northern zone similarly increased from 4.0% to 7.2%, consistent with growth toward Carabayllo and Ancón along the Pan‐American Highway North (Figure 6b). Peru's land titling policy has historically formalised existing occupation rather than preventing it, consistent with the informal expansion typology documented for Peruvian cities (Espinoza and Fort 2020). KDE revealed a progressive outward displacement of growth hotspots: high‐density clusters in Carabayllo, San Martín de Porres, San Juan de Lurigancho, and Villa el Salvador in 1990–2000 (Figure 7b) consolidated toward Carabayllo and San Juan de Lurigancho in 2000–2010 (Figure 7c), before shifting to a broader peripheral pattern centred on Carabayllo, Pachacámac, and Ancón by 2010–2020 (Figure 7d).

**TABLE 5:** Urban expansion (km^2^) and proportional contribution by latitudinal zone (north, centre, south) per inter‐decadal period in the department of Lima.

Zone	1990–2000 km^2^	% period	2000–2010 km^2^	% period	2010–2020 km^2^/% period
North	6.62	4.0	7.35	5.9	9.42/7.2
Centre	155.6	93.4	112.2	89.3	110.0/84.0
South	4.45	2.7	6.03	4.8	11.6/8.8

**FIGURE 6:**
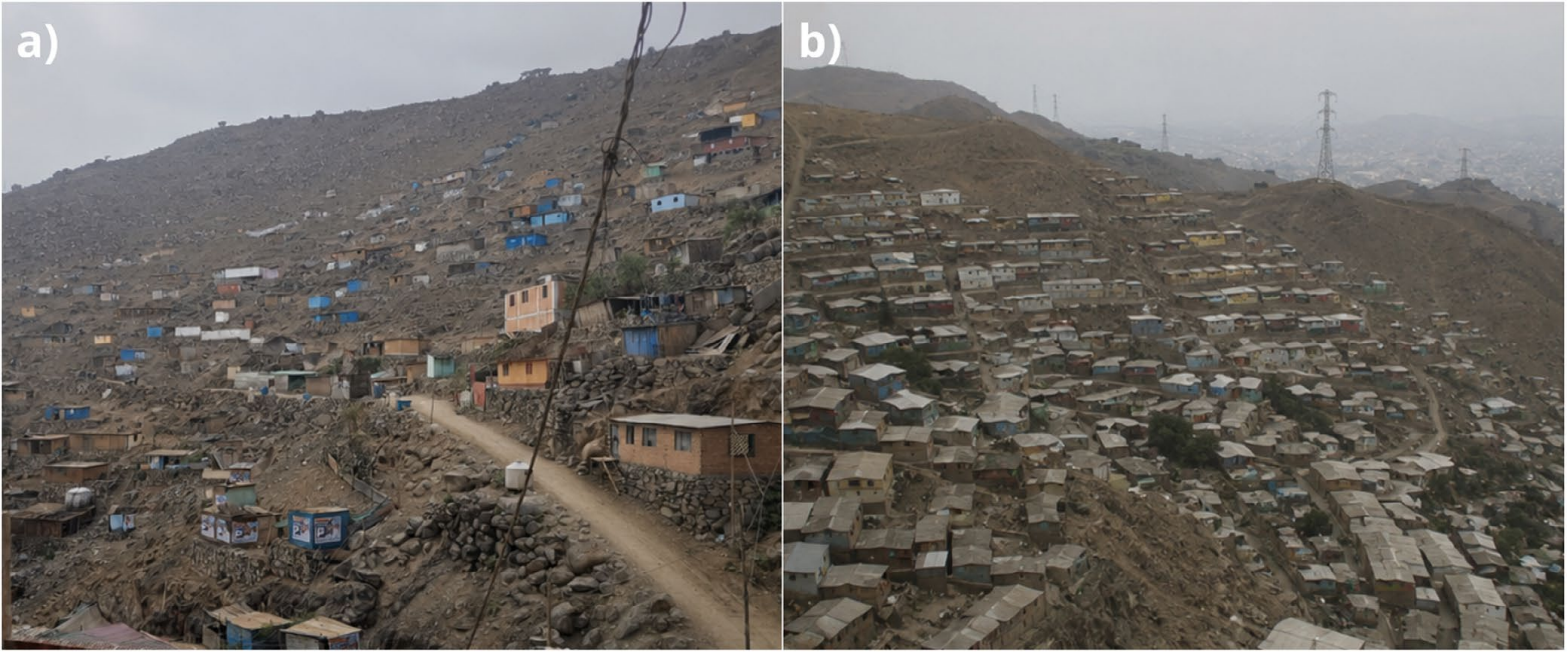
Informal periurban settlements adjacent to (a) Lomas de Villa María, southern sector of metropolitan Lima, and (b) Lomas de Amancaes, northern sector, illustrating the urban encroachment front on lomas ecosystems. Photo credit: Jhade R. Gutierrez‐Gutierrez.

**FIGURE 7:**
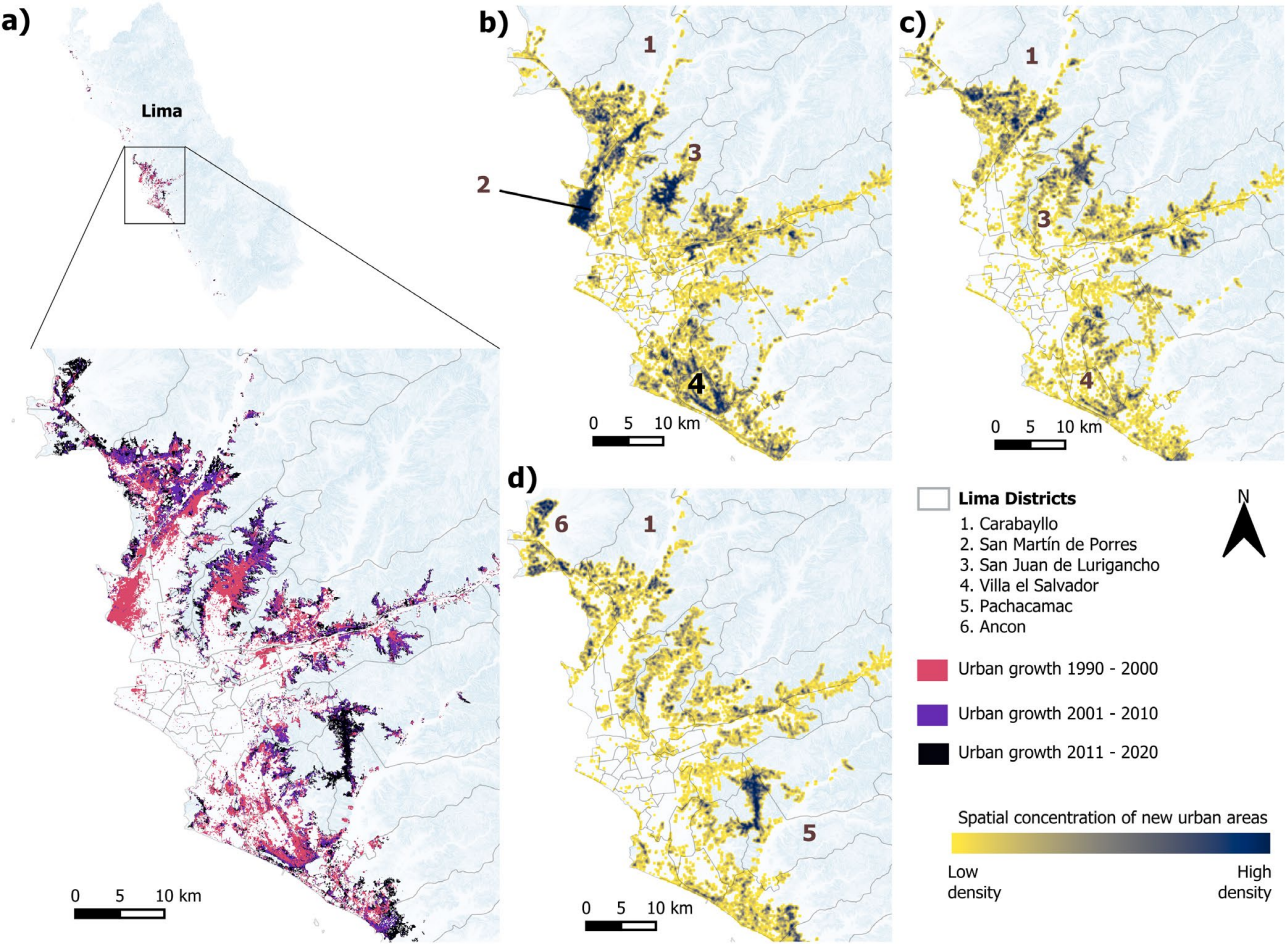
Urban chronology and kernel density estimation of new urban pixels in the department of Lima. (a) Chronological sequence of urbanisation showing the earliest decade of urban pixel acquisition across the department; spatial concentration of new urban areas in (b) 1990–2000, (c) 2000–2010, and (d) 2010–2020.

Landscape metrics revealed a consolidation trajectory rather than classic sprawl (Tables S7 and S8 in Supporting Information): LSI declined from 63.97 to 55.96 (Δ = −8.01) and CI increased from 51.21 to 58.56 (Δ = +7.35), indicating progressive compaction of urban form. Patch number decreased from 1615 to 1051 as mean patch size tripled (0.0027 to 0.0081 ha), reflecting coalescence of isolated urban fragments. The Fractal Dimension Index remained stable (≈0.905), suggesting no change in internal patch complexity. Consolidation was strongest in 2010–2020 (LSI: Δ = −5.01; CI: Δ = +4.82).

### 3.5 Multi‐Temporal Dynamics of Urban Expansion

LCM projections indicated sustained urban expansion from 843.10 km^2^ in 2020 (2.41% of the department) to 1285.75 km^2^ by 2050 (3.67%) and 1563.07 km^2^ by 2070 (4.46%), representing a cumulative gain of 719.97 km^2^ (+85.4% relative to 2020; Figures 8 and S2). Expansion was fastest between 2020 and 2050 (+443.08 km^2^; +52.6%), decelerating in the subsequent two decades (+277.42 km^2^; +21.6%), consistent with the consolidation trend identified in Section 3.4. This single trajectory reflects one deterministic realisation of the calibrated LCM model rather than a probabilistic envelope; its plausibility rests on the Kappa validation reported in Section 2.4.2. Spatially, growth concentrated at the metropolitan fringe of Lima Metropolitana and Callao, with notable expansion toward the southern periurban districts and the lower slopes of the coastal hillsides (Figure 8c–f). Much of this remaining growth targets steep hillside terrain, where construction is costlier and more hazardous, so projected totals may overstate feasible expansion.

**FIGURE 8:**
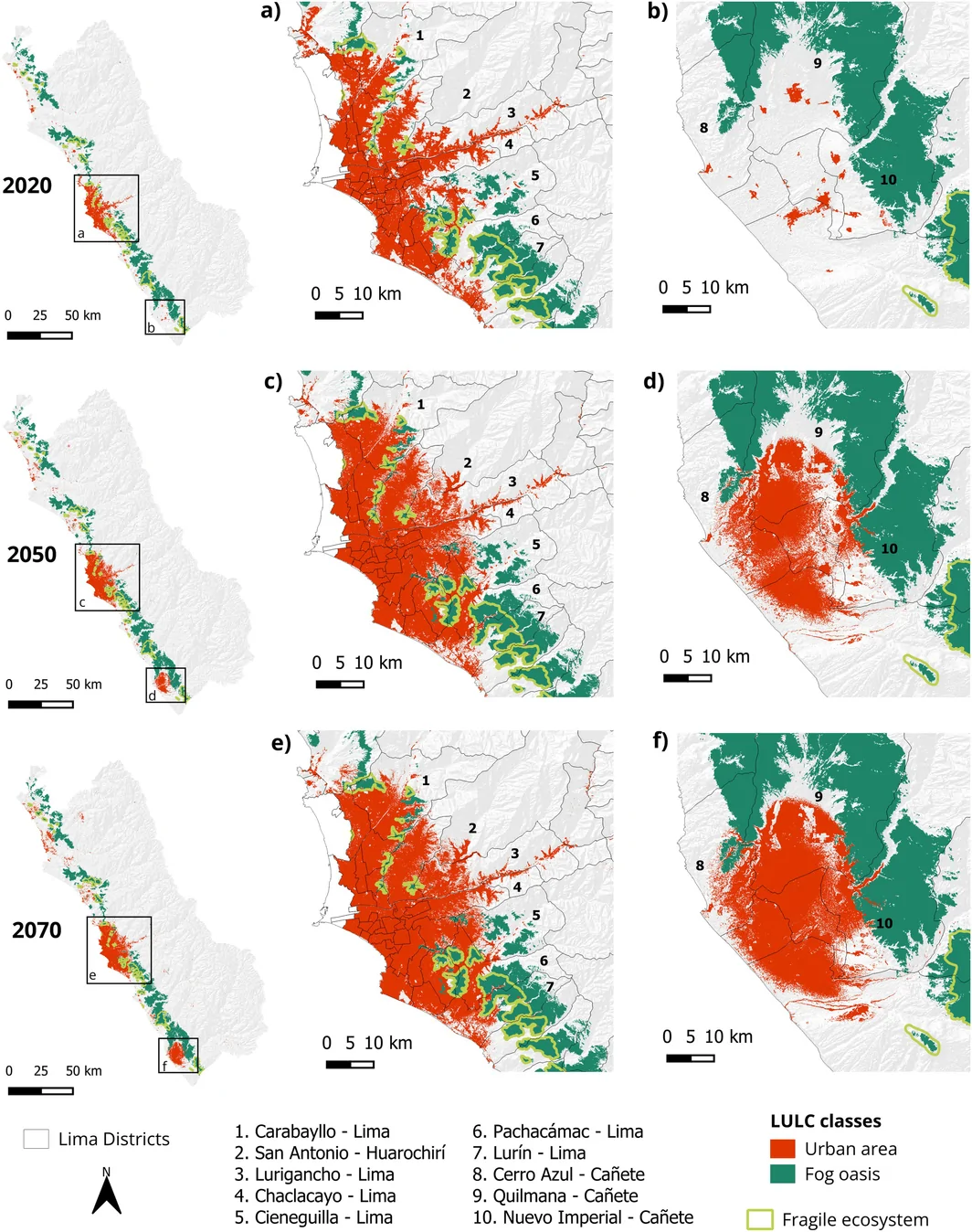
Urban expansion and fog oasis distribution in the department of Lima for the observed baseline (2020) and projected scenarios (2050 and 2070). Left panels show the departmental overview; central panels (a, c, e) detail the Lima Metropolitana and Callao metropolitan area with key districts of urban growth; right panels (b, d, f) show the southern coastal zone highlighting the interface between urban expansion and fog oasis and fragile ecosystem boundaries.

### 3.6 Spatial Overlap Between Suitable Habitat and Urban Expansion

Under current conditions (2020), 36.12 km^2^ of high‐suitability habitat coincide with urban land cover classified as critical conflict zones, while 2855.16 km^2^ remain preserved (Figure 9a, Table S9). By 2050, critical conflict on high‐suitability habitat nearly triples to 99.69 km^2^, with preserved high‐suitability area declining to 2791.59 km^2^, as urban expansion penetrates the metropolitan fringe (Figure 9b, Table S9). By 2070, encroachment on high‐suitability habitat escalates to 126.94 km^2^ (+251% relative to 2020), reducing preserved area to 2764.34 km^2^ (Figure 9c, Table S9). Across all time horizons, the southern periurban front concentrates the largest critical conflict zones, coinciding with the expansion hotspots documented in Section 3.4. This southern periurban front spans the districts of Villa María del Triunfo, Pachacámac, and Lurín in Lima province, and extends into Cerro Azul, Quilmana, and Nuevo Imperial in neighbouring Cañete province (Figure 9), consistent with the informal, land‐trafficking‐driven settlement dynamics already documented for this corridor (García et al. 2014; Piedra Jauregui 2020). The critical‐conflict classification captures where urban land cover overlaps suitable habitat but does not by itself distinguish land‐tenure status; qualitatively, the areas of largest overlap coincide with districts where informal, non‐titled settlement has historically outpaced formal urban planning (Espinoza and Fort 2020; Piedra Jauregui 2020), suggesting that much of this encroachment is not amenable to conventional zoning enforcement alone. Within the protected area framework, high‐suitability habitat inside RCAs and NPAs remains relatively stable across scenarios (RCA: 21.87–23.62 km^2^; NPA: 22.43–54.42 km^2^); however, the majority of high‐suitability habitat at risk lies outside any protected boundary, underscoring the limited buffering capacity of the current conservation framework (Tables S10–S14).

**FIGURE 9:**
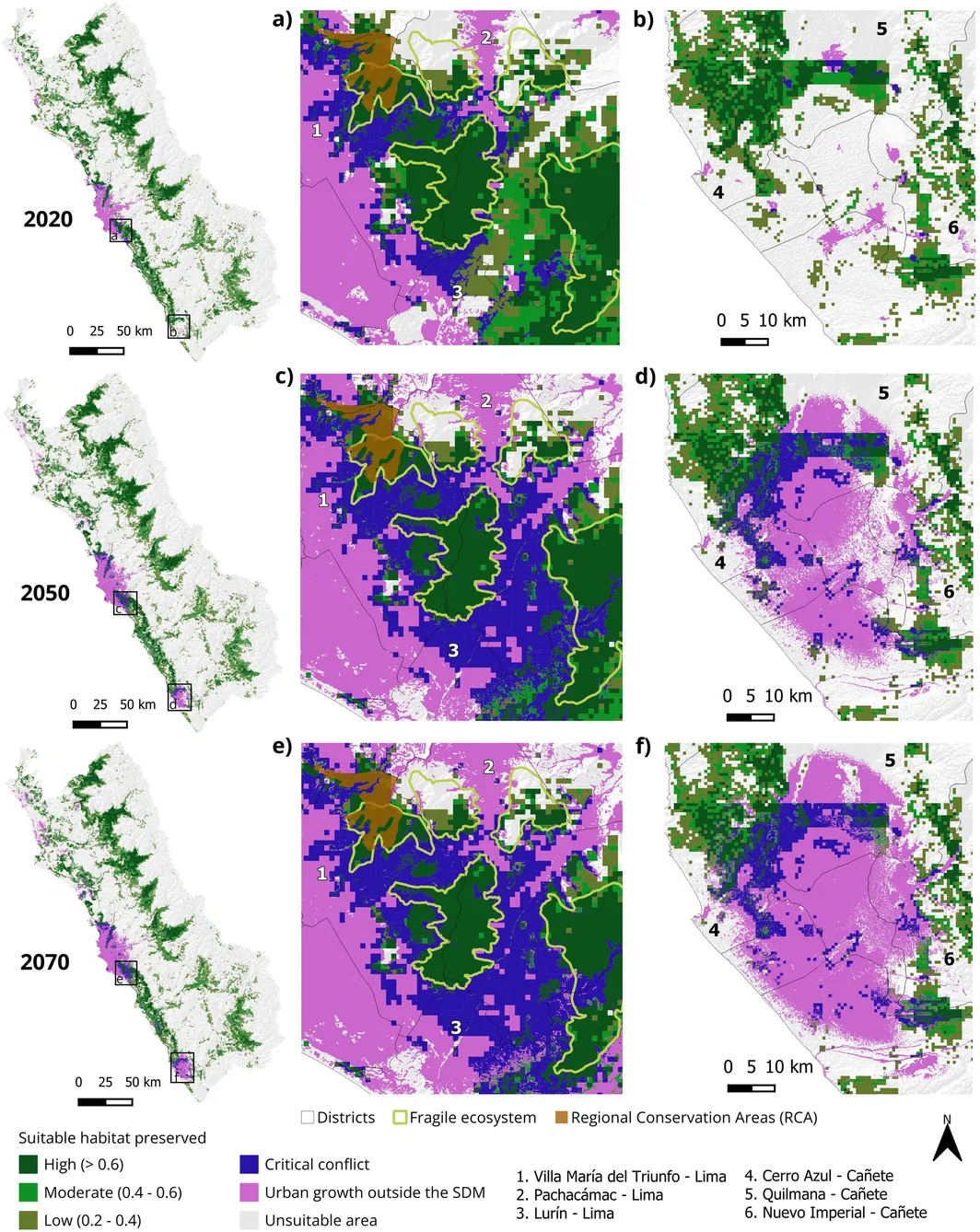
Spatial overlap between habitat suitability for *Vasconcellea candicans* and urban expansion in the department of Lima for (a, b) 2020, (c, d) 2050, and (e, f) 2070. Left panels (a, c, e) detail the metropolitan Lima front, encompassing (1) Villa María del Triunfo, (2) Pachacámac, and (3) Lurín, with Regional Conservation Area (dark orange) and fragile ecosystem (yellow green outline) boundaries overlaid; right panels (b, d, f) detail the southern periurban front extending into Cañete province, encompassing (4) Cerro azul, (5) Quilmana, and (6) Nuevo Imperial. Green shades indicate preserved suitable habitat (high: > 0.6; moderate: 0.4–0.6; low: 0.2–0.4); blue denotes critical conflict zones where urban growth overlaps suitable habitat; purple indicates urban expansion outside the SDM envelope; grey indicates unsuitable area.

## 4 Discussion

### 4.1 Model Performance and Key Environmental Drivers

All five retained algorithms achieved excellent discriminative performance (AUC > 0.90, TSS > 0.70). Their convergence on a common predictor hierarchy strengthens confidence in the identified drivers and reduces single‐algorithm uncertainty (Araújo and New 2007; Grenouillet et al. 2011; Hao et al. 2019). Random Forest achieved the highest AUC (0.968), consistent with its documented superiority in ensemble SDM frameworks, where resistance to overfitting and capacity to capture non‐linear species‐environment relationships confer a systematic advantage over regression‐based methods (Mi et al. 2017; Zhang et al. 2020). This robust performance is particularly evident in studies with complex topographical constraints or limited occurrence records, where decision‐tree architectures outperform simpler models by minimising bias through recursive partitioning (Valavi et al. 2022; Wisz et al. 2008). MaxEnt's comparatively lower AUC likely reflects its use of default, untuned regularisation settings, since MaxEnt performance is particularly sensitive to such parameterisation (Ahmadi et al. 2023), whereas Random Forest is documented to remain robust under default settings even with small samples (Mi et al. 2017).

Bio07 (Temperature Annual Range) emerged as the dominant climatic predictor, reflecting the narrow thermal envelope imposed by the inversion layer generated by the subtropical anticyclone and the Humboldt Current, which governs fog incidence and vegetation phenology across Lima's *lomas* (Haas and Dillon 2003; Manrique et al. 2014). In these ecosystems, thermal seasonality acts as a critical filter for plant survival, as extreme fluctuations in the annual range disrupt the balance between atmospheric moisture interception and evapotranspirative demand, a physiological constraint common to fog‐dependent flora in the Atacama‐Sechura corridor (Pinto et al. 2006; Schulz et al. 2011). The importance of silt content, cation exchange capacity, and total nitrogen reflects the fine‐textured, organically active soils of fog‐influenced hillsides, where atmospheric water deposition drives pedogenic processes (Camel et al. 2024; Eusebio and Mendoza 2006). These predictors point to concrete management actions: protecting fog‐influenced topsoil from compaction and organic‐matter loss, and, where degradation has already occurred, applying organic amendments to restore fertility, an approach already tested in Lima's lomas (Flores and Van Meerbeek 2024). Similar patterns have been reported in the Lomas de Amancaes, where fog‐derived moisture sustains soil carbon accumulation and nutrient cycling under desert conditions (Guerrero‐Palomino et al. 2022). In addition, the contribution of terrain aspect (2.4%) confirms that terrain orientation is secondary to soil conditions and thermal seasonality within the narrow altitudinal band occupied by *V. candicans* (Mod et al. 2016). Elevation was excluded during VIF filtering, likely reflecting collinearity with bio07 in this narrow coastal gradient. This is consistent with fog‐dependent systems elsewhere on the Pacific coast, where fog‐water interception is governed primarily by wind direction and slope rather than aspect (Borthagaray et al. 2010), so microclimatic variation across the narrow lomas belt may already be captured by bio07 and slope, leaving aspect comparatively uninformative.

### 4.2 Current and Projected Habitat Suitability

The ensemble identified 8500.42 km^2^ of suitable habitat across Lima, yet high‐suitability areas accounted for only 8.26% of this total, a spatial restriction consistent with the narrow climatic and edaphic conditions defining functional *lomas* habitat (Moat et al. 2021). Fragile ecosystems and fog oases concentrated disproportionate fractions of high‐suitability habitat (46.85% and 25.01% of their respective areas). This pattern mirrors findings for other endemic plants in fragmented desert ecosystems, where core suitable habitat is restricted to microenvironmentally stable refugia (Muenchow et al. 2013; Pauca‐Tanco et al. 2020). For *V. candicans* as a Critically Endangered species, the loss of even small high‐suitability patches entails community‐level consequences disproportionate to their area (Seoane Byrne and Jeri Kukulis 2018; Villasante Benavides et al. 2021).

Future projections diverged substantially between bounding scenarios, from −4.08% under IPSL‐CM6A‐LR SSP2‐4.5 to +56.35% under ACCESS‐CM2 SSP5‐8.5 by 2070, reflecting the high sensitivity of *lomas* systems to thermal seasonality and precipitation variability (Goberville et al. 2015; Wiens et al. 2009). The projected expansion under SSP5‐8.5 may partly reflect an upward altitudinal shift in suitable conditions, consistent with the documented tendency of plant species in thermally constrained ecosystems to track suitable climates toward higher elevations as temperatures rise (Guerrero et al. 2011; Pauca‐Tanco et al. 2020). However, high‐emissions trajectories also intensify ENSO variability and disrupt the Humboldt Current dynamics sustaining fog formation, a processes not fully captured by bioclimatic variables alone (Beresford‐Jones et al. 2015; Haas and Dillon 2003), and causing the model to overestimate realised habitat gains. ACCESS‐CM2 also carries a documented larger energy imbalance at the top of the atmosphere than other CMIP6 models (Bi et al. 2020), which may inflate its projected signal; its SSP5‐8.5 gain is best read as an upper bound, not the most likely outcome. Similar scenario divergence has been reported for range‐restricted South American flora, where emission trajectory exerts greater influence on projected outcomes than modelling algorithm choice even in páramo (Rosero‐Erazo et al. 2024), and native shrubs in Andean arid ecosystems (Atauchi et al. 2018). Moreover, an increase in projected suitability does not guarantee colonisation: SDMs generally predict occurrence better than realised population outcomes, since dispersal, establishment, and phenological constraints are excluded from the modelled niche (Lee‐Yaw et al. 2022). For *V. candicans* , projected gains under SSP5‐8.5 should therefore be read as an upper bound on climatically available habitat, not an expectation of realised range expansion. Under both scenarios, the fastest rates of projected change concentrate in the 2020–2050 window, defining the most critical management horizon before urban consolidation renders conservation intervention structurally impractical.

### 4.3 Spatial Overlap Between Urban Expansion and Suitable Habitat

Urban encroachment constitutes the most immediate and structurally irreversible threat to *V. candicans* habitat: unlike climate‐driven suitability shifts, urbanised land is permanently removed from ecological function (Flood Chávez and Niewiadomski 2022). The 36.12 km^2^ of high‐suitability habitat already coinciding with urban land in 2020 represents a non‐recoverable baseline loss, escalating to 126.94 km^2^ by 2070 under the low‐projection scenario, consistent with Table S9, a 251% increase concentrated along the periurban southern front (Lurín, Pachacámac, Villa María del Triunfo), consistent with the outward displacement of Lima's expansion hotspots toward coastal hillside districts (Ramirez Herrera et al. 2022). This expansion outpaces population growth itself, which decelerated from 2.0% to 1.2% annually between 1993 and 2017 (INEI), pointing to densification and informal occupation rather than demographic pressure alone as drivers. Land tenure compounds this risk, as the largest conflict zones coincide with districts where informal, non‐titled settlement has historically outpaced formal planning, so zoning enforcement alone is unlikely to contain encroachment. Beyond direct habitat conversion, urban proximity likely degrades adjacent suitable habitat through edge effects such as altered microclimate, dust, and pollution spreading from the built margin (Romero et al. 2020; de Farias et al. 2021), so suitability near the fringe may overstate effective habitat quality. This encroachment pattern is a recognised dynamic in Lima, where unmanaged urban sprawl persistently pressures remnant natural areas and their edges (García et al. 2014; Piedra Jauregui 2020). The landscape consolidation trajectory, marked by decreasing LSI, increasing CI, and coalescence of urban patches, implies a progressive loss of connectivity between *lomas* fragments, constraining dispersal for species with restricted ranges (Nieuwland and Mamani 2017; Soria Dall'Orso and Romo Román 2019).

The compounding interaction between urban expansion and climate change is systematically underestimated by single‐threat assessments. Under SSP5‐8.5, projected gains in high‐suitability area are partly offset by simultaneous urban encroachment into newly suitable areas, a direct synergy in which climate‐driven habitat gain and urban‐driven habitat loss act on the same emerging areas rather than independently, underscoring the need for spatially integrated multi‐threat assessments that jointly quantify climatic and anthropogenic pressures, an approach previously applied to mid‐sized Peruvian cities (Barboza et al. 2025, 2026). Integrating SDMs with land‐use projections is essential to reveal the true conservation status of threatened plants, as evaluating climatic shifts without accounting for concurrent landscape anthropisation leads to overly optimistic scenarios (Nzei et al. 2022; Ramachandran et al. 2020). Conservation planning must therefore account for both threat dimensions simultaneously, prioritising the 2020–2050 window before irreversible urban consolidation forecloses management options.

### 4.4 Protected Area and Conservation Priorities

RCAs concentrate the highest within‐protected‐area suitability for *V. candicans* (21.12 km^2^ currently, rising to 23.62 km^2^ by 2070 under ACCESS‐CM2 SSP5‐8.5). Yet they also bear the greatest projected urban exposure among formally protected units, reflecting their location at the interface between the metropolitan fringe and the most ecologically valuable *lomas* fragments. NPAs, by contrast, show negligible intersection with the species' core range (1.18% classified as high suitability), revealing a spatial mismatch between protection boundaries and the species' distributional core. Suitability and conflict figures for RCAs and NPAs are reported at the aggregate scale relevant to national conservation policy (Tables S10–S14). Such representation gaps are characteristic of South American drylands, where protected area networks are underrepresenting restricted‐range endemics in highly pressured arid environments and in tropical Andes (Bax and Francesconi 2019; Fajardo et al. 2014; Joppa and Pfaff 2009; Tognelli et al. 2008). This mismatch is also a connectivity problem: isolated RCAs and NPAs do little to maintain dispersal between lomas fragments unless the intervening unprotected patches are also secured.

A high‐suitability habitat exists outside any protected boundary, concentrated along the periurban southern and central expansion fronts. Priority extension targets include the fragments bordering Villa María del Triunfo, Pachacámac, and Lurín, which would also close current connectivity gaps between isolated lomas patches. Where formal designation lags, private conservation areas (ACPs) offer a faster route, as already shown at ACP Lomas Cerro Campana (Pollack et al. 2020); urban growth boundaries could be operationalised by fixing existing fragile‐ecosystem and RCA polygons as no‐build limits. Therefore, effective conservation will require instruments beyond formal designation: buffer zone regulations, urban growth boundaries aligned with *lomas* limits, and integration of high‐suitability mapping into Lima's metropolitan spatial planning framework (Contreras Zuloaga 2023; Guerrero‐Palomino et al. 2022; MINAM 2020). Extending the RCA network to unprotected high‐suitability patches identified here represents the most spatially explicit conservation priority, consistent with regional evidence that integrating SDMs into systematic conservation planning is a cost‐effective tool for guiding reserve network expansion across threatened Peruvian ecosystems (Fajardo et al. 2014; Villero et al. 2017).

### 4.5 Limitations

This study represents the first spatially integrated assessment combining ensemble SDMs with urban growth projections for a Critically Endangered *lomas* endemic in Lima. Nevertheless, this study is not free from limitations, divided in two groups: those affecting the habitat suitability estimates, and those affecting the urban growth projections.

For habitat suitability, limited presence records, although subjected to spatial thinning to reduce sampling bias (Boria et al. 2014; Veloz 2009), may still underestimate the species' actual distribution, particularly in poorly surveyed or inaccessible *lomas* fragments. Reliance on GBIF, which aggregates but does not fully capture records held in regional databases or unpublished literature, is a related constraint, as further digitisation of such sources is known to fill persisting spatial gaps in GBIF‐based inventories (Blades et al. 2025). The 30‐year bioclimatic baseline (1970–2000) also predates most occurrence records (86% collected in 2020–2025), a temporal mismatch known to reduce model accuracy (Roubicek et al. 2010) and, combined with the small sample, raises the risk of overfitting despite the favourable AUC/TSS scores reported. The 2050 and 2070 suitability surfaces likewise remain unvalidated against any independent future observation, since none yet exists; their reliability rests on current model performance and on the plausibility of the GCM × SSP envelope rather than on direct verification. Therefore, future research should incorporate systematic field surveys and locally validated occurrence records to improve model calibration and reduce uncertainty in predicted suitability patterns. Topographic and edaphic predictors were held constant across future projections, a common assumption in ensemble SDMs (Thuiller et al. 2009), but one that may overlook progressive soil degradation associated with informal urbanisation and land‐use disturbance: such processes could intensify habitat degradation and lead to greater suitability losses than those estimated here (Camel et al. 2024).

Related to the urban projection and its integration with suitability, LCM projections assume the continuity of historical land‐cover transitions, although future changes in urban policy, RCA enforcement, infrastructure development, or informal land trafficking could alter urban expansion trajectories (Espinoza and Fort 2020; Li and Gong 2016). More broadly, the exclusion of biotic interactions, demographic processes, and dispersal constraints, relevant to both dimensions, may limit fine‐scale predictive accuracy under novel climatic and urbanisation scenarios (Elith and Leathwick 2009; Wiens et al. 2009). Transferring the framework to other lomas units would also require re‐calibration, as predictor importance can shift with grain size and geographic extent (Manzoor et al. 2018). Future research should therefore combine expanded field surveys, dynamic environmental predictors, dispersal data, and socio‐economic land‐use scenarios to refine vulnerability assessments for *lomas* endemic species.

## 5 Conclusions

The integration of ensemble SDMs with spatially explicit urban growth projections reveals that *V. candicans* faces a dual and compounding threat in Lima's *lomas*: a climatically sensitive and edaphically constrained distribution concentrated in fragile ecosystems, and an accelerating urban expansion that irreversibly eliminates high‐suitability habitat at the metropolitan fringe. The 2020–2050 window offers the greatest opportunity for effective intervention, before urban consolidation forecloses it. Climate projections introduce substantial uncertainty, but even under moderate emissions scenarios suitable habitat contracts, and apparent gains under high emissions are likely overstated.

Effective conservation of *V. candicans* requires moving beyond single‐threat frameworks and formal protected area designation alone. Extending the Regional Conservation Area network to unprotected high‐suitability patches, prioritising the fragments bordering Villa María del Triunfo, Pachacámac, and Lurín, combined with urban growth boundaries aligned with *lomas* limits and integration of habitat suitability mapping into metropolitan spatial planning. Together, these measures represents the most operationally tractable response to the encroachment front documented here. More broadly, the spatially integrated approach demonstrated in this study is directly transferable to other endemic taxa inhabiting ecosystems at the urban–desert interface across coastal South America, where rapid urbanisation and climate change are converging as the dominant drivers of biodiversity loss. Priorities for future work include field validation of the named patches, incorporation of dispersal data, and extension of this framework to other lomas systems.

## Author Contributions

**Rebeca M. Cruz‐Zegarra:** conceptualization (lead), data curation (lead), formal analysis (lead), investigation (lead), methodology (lead), validation (equal), visualization (lead), writing – original draft (lead). **Jhade R. Gutierrez‐Gutierrez:** conceptualization (supporting), formal analysis (equal), investigation (equal), methodology (supporting), writing – review and editing (supporting). **Abner S. Rivera‐Fernandez:** conceptualization (supporting), investigation (supporting), methodology (supporting), writing – review and editing (supporting). **Alexander Cotrina‐Sanchez:** conceptualization (lead), supervision (lead), writing – original draft (supporting), writing – review and editing (equal). **Samuel Astete:** conceptualization (lead), investigation (equal), methodology (supporting), resources (lead), supervision (lead), writing – review and editing (equal).

## Funding

This research was supported by Universidad Peruana de Ciencias Aplicadas, grant UPC‐Expost‐2026‐2.

## Conflicts of Interest

The authors declare no conflicts of interest.

## Supporting information


**Table S1:** Summary of *Vasconcellea candicans* occurrence records (*n* = 101) used for ensemble species distribution modelling.
**Table S2:** Full list of the 101 *Vasconcellea candicans* occurrence records (post spatial‐thinning) used to calibrate the ensemble SDM, with associated coordinates, temporal, and provenance metadata.
**Table S3:** Full list of the 31 environmental variables initially considered for ensemble species distribution modelling of *Vasconcellea candicans*.
**Table S4:** Combined high‐ and moderate‐suitability ‘Area of Interest’ (AOI) for *Vasconcellea candicans* under current conditions, by spatial unit.
**Table S5:** Projected habitat suitability of *Vasconcellea candicans* across all GCM–SSP combinations for 2050 and 2070 in the department of Lima. Values in parentheses indicate percentage change relative to current conditions (1970–2000). Suitability classes are defined as: Low (0.2–0.4), Moderate (0.4–0.6), and High (> 0.6). Percentages are calculated relative to the total surface area of the department (35,892.49 km^2^).
**Table S6:** Range shift statistics for *Vasconcellea candicans* under future climate change scenarios in comparison to current climate conditions.
**Table S7:** Landscape metrics of urban expansion in the department of Lima for 1990, 2000, 2010, and 2020. LSI: Landscape Shape Index; FRAC: Fractal Dimension Index; CI: Compactness Index; NP: number of patches; MPS: mean patch size (ha).
**Table S8:** Inter‐decadal changes (Δ) in landscape metrics of urban expansion in the department of Lima for the periods 1990–2000, 2000–2010, and 2010–2020, relative to the preceding time step.
**Table S9:** Spatial overlap between suitable habitat and urban expansion.
**Table S10:** Habitat suitability distribution (km^2^) for *Vasconcellea candicans* across key spatial units under current conditions (2020).
**Table S11:** Habitat suitability distribution (km^2^) for *Vasconcellea candicans* across key spatial units under IPSL‐CM6A‐LR SSP2‐4.5 for 2050.
**Table S12:** Habitat suitability distribution (km^2^) for *Vasconcellea candicans* across key spatial units under ACCESS‐CM2 SSP5‐8.5 for 2050.
**Table S13:** Habitat suitability distribution (km^2^) for *Vasconcellea candicans* across key spatial units under IPSL‐CM6A‐LR SSP2‐4.5 for 2070.
**Table S14:** Habitat suitability distribution (km^2^) for *Vasconcellea candicans* across key spatial units under ACCESS‐CM2 SSP5‐8.5 for 2070.
**Figure S1:** Projected habitat suitability of *Vasconcellea candicans* in the department of Lima across all six GCM–SSP combinations for 2050 and 2070. Upper panels (a–f): 2050s projections under ACCESS‐CM2 SSP2‐4.5 (a), ACCESS‐CM2 SSP5‐8.5 (b), IPSL‐CM6A‐LR SSP2‐4.5 (c), IPSL‐CM6A‐LR SSP5‐8.5 (d), MIROC6 SSP2‐4.5 (e), and MIROC6 SSP5‐8.5 (f). Lower panels (a–f): corresponding projections for the 2070s. Suitability classes Low: 0.2–0.4; Moderate: 0.4–0.6; High: > 0.6.
**Figure S2:** Land use and land cover distribution in the department of Lima for observed years (a) 1990, (b) 2000, (c) 2010, and (d) 2020, and projected scenarios for (e) 2050 and (f) 2070, derived from the Land Change Modeller (LCM). Urban area and fog oasis are shown as primary land cover classes.

## Data Availability

The datasets used in this study are publicly available at https://doi.org/10.6084/m9.figshare.32328792.
