# Supplementary material for: Assessing Habitat Suitability and Exposure of the Endemic Vasconcellea candicans Under Climate Change and Urban Growth in Lima, Peru
Source: Ecol Evol. 2026 Oct 1;16(10):e74437. doi: 10.1002/ece3.74437 (PMC13628455; doi:10.1002/ece3.74437)
Supplement: Supplementary file 1 [file ECE3-16-e74437-s001.docx]

**Table S1.** Summary of *Vasconcellea candicans* occurrence records (n = 101) used for ensemble species distribution modelling.

A) **Temporal distribution of records**

| **Period** | **N records** | **% of total** |
| --- | --- | --- |
| 1960–1999 | 3 | 3.00% |
| 2000–2019 | 8 | 7.90% |
| 2020–2025 | 87 | 86.10% |
| Undated | 3 | 3.00% |
| **Total** | 101 | 100.00% |

B) **Data source type of occurrence records (n = 101)**

| **Source** | **Type** | **N records** | **% of total** |
| --- | --- | --- | --- |
| iNaturalist | Citizen-science observation | 94 | 93.10% |
| CUVC (Universidad del Valle Herbarium) | Herbarium specimen | 2 | 2.00% |
| Bioversity International | Genebank/field collection record | 2 | 2.00% |
| MNHN (Muséum national d'Histoire Naturelle) | Herbarium specimen | 1 | 1.00% |
| MO/F (Missouri Botanical Garden / Field Museum) | Herbarium specimen | 1 | 1.00% |
| Herbarium of South China Botanical Garden | DNA-barcoding voucher specimen | 1 | 1.00% |
| Total |  | 101 | 100.00% |

C) **Seasonal distribution of occurrence records (n = 101)**

| **Season** | **Months** | **N records** | **% of total** |
| --- | --- | --- | --- |
| Fog season | May–October | 66 | 65.30% |
| Dry season | November–April | 32 | 31.70% |
| Undated | — | 3 | 3.00% |
| Total |  | 101 | 100.00% |

D) **Coordinate uncertainty (coordinateUncertaintyInMeters) of occurrence records**

| **Metric** | **Value** |
| --- | --- |
| Records with reported uncertainty | 81 of 101 (80.20%) |
| Records missing this field | 20 of 101 (19.80%) |
| Median uncertainty (of reported) | 139 m |
| Range (of reported) | 1–12,201 m |
| Records exceeding 250 m grid resolution (of reported) | 26 of 81 (32.10%) |
| Note | No explicit threshold on reported coordinate uncertainty was applied; this is partly offset by the spatial thinning procedure (Section 2.3.1), which aggregates nearby low-precision points into shared 250 m grid cells, and by the 10-partition ensemble approach (Section 2.3.3), which reduces sensitivity to any single record. Phenological stage (flowering/fruiting) was not recorded for any occurrence. |

**Table S2.** Full list of the 101 *Vasconcellea candicans* occurrence records (post spatial-thinning) used to calibrate the ensemble SDM, with associated coordinates, temporal, and provenance metadata.

| **ID** | **Longitude** | **Latitude** | **Year** | **Month** | **Season** | **Source (institutionCode)** | **Source type** | **basisOfRecord** | **Locality** | **Coord. uncertainty (m)** |
| --- | --- | --- | --- | --- | --- | --- | --- | --- | --- | --- |
| 1 | -76.8879281 | -10.80561 | 2023 | 11 | Dry season (Nov-Apr) | iNaturalist | Citizen-science observation | HUMAN_OBSERVATION | — | 50 |
| 2 | -76.7778844 | -11.3176497 | 2023 | 8 | Fog season (May-Oct) | iNaturalist | Citizen-science observation | HUMAN_OBSERVATION | — | 365 |
| 3 | -77.5010282 | -11.3333702 | 1989 | 10 | Fog season (May-Oct) | CUVC | Field/genebank record | OCCURRENCE | Lomas de Lachay. | — |
| 4 | -77.366281 | -11.3580739 | 2024 | 7 | Fog season (May-Oct) | iNaturalist | Citizen-science observation | HUMAN_OBSERVATION | — | 5 |
| 5 | -77.3595436 | -11.3580739 | 2025 | 6 | Fog season (May-Oct) | iNaturalist | Citizen-science observation | HUMAN_OBSERVATION | — | 166 |
| 6 | -77.366281 | -11.3603197 | 2022 | 3 | Dry season (Nov-Apr) | iNaturalist | Citizen-science observation | HUMAN_OBSERVATION | — | 98 |
| 7 | -77.3640352 | -11.3603197 | 2025 | 3 | Dry season (Nov-Apr) | iNaturalist | Citizen-science observation | HUMAN_OBSERVATION | — | — |
| 8 | -77.3617894 | -11.3603197 | 2025 | 10 | Fog season (May-Oct) | iNaturalist | Citizen-science observation | HUMAN_OBSERVATION | — | 11 |
| 9 | -77.2292879 | -11.4007438 | — | — | Undated | Bioversity | Field/genebank record | OCCURRENCE | — | — |
| 10 | -77.2270421 | -11.409727 | 2017 | 9 | Fog season (May-Oct) | Herbarium of South China Botanical Garden | DNA-barcoding voucher | MATERIAL_SAMPLE | Lomas de Iguanil | — |
| 11 | -76.6588577 | -11.4883296 | 2025 | 11 | Dry season (Nov-Apr) | iNaturalist | Citizen-science observation | HUMAN_OBSERVATION | — | — |
| 12 | -76.6925445 | -11.5107875 | 2021 | 2 | Dry season (Nov-Apr) | iNaturalist | Citizen-science observation | HUMAN_OBSERVATION | — | 11 |
| 13 | -76.6947903 | -11.5130333 | 2024 | 11 | Dry season (Nov-Apr) | iNaturalist | Citizen-science observation | HUMAN_OBSERVATION | — | 4 |
| 14 | -76.5735177 | -11.6971879 | 2024 | 9 | Fog season (May-Oct) | iNaturalist | Citizen-science observation | HUMAN_OBSERVATION | — | 3995 |
| 15 | -76.6094503 | -11.7151542 | 2023 | 7 | Fog season (May-Oct) | iNaturalist | Citizen-science observation | HUMAN_OBSERVATION | — | 10 |
| 16 | -76.6094503 | -11.7398579 | 2024 | 2 | Dry season (Nov-Apr) | iNaturalist | Citizen-science observation | HUMAN_OBSERVATION | — | 122 |
| 17 | -76.6094503 | -11.7421037 | 2022 | 8 | Fog season (May-Oct) | iNaturalist | Citizen-science observation | HUMAN_OBSERVATION | — | 4 |
| 18 | -76.6094503 | -11.7443494 | 2018 | 10 | Fog season (May-Oct) | iNaturalist | Citizen-science observation | HUMAN_OBSERVATION | — | 5 |
| 19 | -76.6072045 | -11.7443494 | 2018 | 10 | Fog season (May-Oct) | iNaturalist | Citizen-science observation | HUMAN_OBSERVATION | — | 5 |
| 20 | -76.6004672 | -11.7443494 | 2025 | 1 | Dry season (Nov-Apr) | iNaturalist | Citizen-science observation | HUMAN_OBSERVATION | — | — |
| 21 | -76.6049587 | -11.7465952 | 2024 | 12 | Dry season (Nov-Apr) | iNaturalist | Citizen-science observation | HUMAN_OBSERVATION | — | 4 |
| 22 | -76.6206793 | -11.7510868 | 2018 | 4 | Dry season (Nov-Apr) | iNaturalist | Citizen-science observation | HUMAN_OBSERVATION | — | 16 |
| 23 | -76.6004672 | -11.7555784 | 2024 | 2 | Dry season (Nov-Apr) | iNaturalist | Citizen-science observation | HUMAN_OBSERVATION | — | — |
| 24 | -77.0383959 | -11.7982484 | 2025 | 9 | Fog season (May-Oct) | iNaturalist | Citizen-science observation | HUMAN_OBSERVATION | — | — |
| 25 | -76.3107605 | -11.8117231 | 2021 | 11 | Dry season (Nov-Apr) | iNaturalist | Citizen-science observation | HUMAN_OBSERVATION | — | 12201 |
| 26 | -76.6094503 | -11.8184605 | 2022 | 9 | Fog season (May-Oct) | iNaturalist | Citizen-science observation | HUMAN_OBSERVATION | — | 209 |
| 27 | -76.3871173 | -11.8431641 | — | — | Undated | MNHN | Herbarium specimen | PRESERVED_SPECIMEN | — | — |
| 28 | -76.400592 | -11.8499015 | 2002 | 10 | Fog season (May-Oct) | iNaturalist | Citizen-science observation | HUMAN_OBSERVATION | — | 99 |
| 29 | -76.3848715 | -11.8499015 | 2024 | 7 | Fog season (May-Oct) | iNaturalist | Citizen-science observation | HUMAN_OBSERVATION | — | 90 |
| 30 | -76.3781341 | -11.8588846 | 2024 | 3 | Dry season (Nov-Apr) | iNaturalist | Citizen-science observation | HUMAN_OBSERVATION | — | 461 |
| 31 | -76.4342788 | -11.8611304 | 2022 | 10 | Fog season (May-Oct) | iNaturalist | Citizen-science observation | HUMAN_OBSERVATION | — | 261 |
| 32 | -76.4297873 | -11.865622 | 2022 | 10 | Fog season (May-Oct) | iNaturalist | Citizen-science observation | HUMAN_OBSERVATION | — | 219 |
| 33 | -76.4275415 | -11.865622 | 2022 | 10 | Fog season (May-Oct) | iNaturalist | Citizen-science observation | HUMAN_OBSERVATION | — | 187 |
| 34 | -76.4702115 | -11.8701136 | 2022 | 6 | Fog season (May-Oct) | iNaturalist | Citizen-science observation | HUMAN_OBSERVATION | — | 15 |
| 35 | -76.4365246 | -11.8880799 | 2023 | 11 | Dry season (Nov-Apr) | iNaturalist | Citizen-science observation | HUMAN_OBSERVATION | — | 165 |
| 36 | -76.4342788 | -11.8880799 | 2024 | 9 | Fog season (May-Oct) | iNaturalist | Citizen-science observation | HUMAN_OBSERVATION | — | 271 |
| 37 | -76.4410162 | -11.8903257 | 2024 | 10 | Fog season (May-Oct) | iNaturalist | Citizen-science observation | HUMAN_OBSERVATION | — | 3 |
| 38 | -76.4387704 | -11.8903257 | 2021 | 10 | Fog season (May-Oct) | iNaturalist | Citizen-science observation | HUMAN_OBSERVATION | — | 204 |
| 39 | -76.4365246 | -11.8903257 | 2021 | 10 | Fog season (May-Oct) | iNaturalist | Citizen-science observation | HUMAN_OBSERVATION | — | 320 |
| 40 | -76.4410162 | -11.8925715 | 2021 | 10 | Fog season (May-Oct) | iNaturalist | Citizen-science observation | HUMAN_OBSERVATION | — | 374 |
| 41 | -76.443262 | -11.8948173 | 2024 | 4 | Dry season (Nov-Apr) | iNaturalist | Citizen-science observation | HUMAN_OBSERVATION | — | 221 |
| 42 | -76.4410162 | -11.8948173 | 2022 | 10 | Fog season (May-Oct) | iNaturalist | Citizen-science observation | HUMAN_OBSERVATION | — | 139 |
| 43 | -76.4387704 | -11.897063 | 2023 | 4 | Dry season (Nov-Apr) | iNaturalist | Citizen-science observation | HUMAN_OBSERVATION | — | 710 |
| 44 | -76.4387704 | -11.8993088 | 2025 | 4 | Dry season (Nov-Apr) | iNaturalist | Citizen-science observation | HUMAN_OBSERVATION | — | 145 |
| 45 | -76.4769488 | -11.9038004 | 2021 | 11 | Dry season (Nov-Apr) | iNaturalist | Citizen-science observation | HUMAN_OBSERVATION | — | 328 |
| 46 | -76.4971609 | -11.9285041 | 2024 | 4 | Dry season (Nov-Apr) | iNaturalist | Citizen-science observation | HUMAN_OBSERVATION | — | 941 |
| 47 | -76.4769488 | -11.9307499 | 2023 | 7 | Fog season (May-Oct) | iNaturalist | Citizen-science observation | HUMAN_OBSERVATION | — | 1309 |
| 48 | -76.474703 | -11.9307499 | 2017 | 5 | Fog season (May-Oct) | iNaturalist | Citizen-science observation | HUMAN_OBSERVATION | — | 1 |
| 49 | -76.4702115 | -11.9374872 | 2023 | 5 | Fog season (May-Oct) | iNaturalist | Citizen-science observation | HUMAN_OBSERVATION | — | 8 |
| 50 | -77.0361501 | -11.9936319 | 2022 | 8 | Fog season (May-Oct) | iNaturalist | Citizen-science observation | HUMAN_OBSERVATION | — | 484 |
| 51 | -77.0316585 | -11.9981235 | 2008 | 9 | Fog season (May-Oct) | iNaturalist | Citizen-science observation | HUMAN_OBSERVATION | — | 31 |
| 52 | -77.0361501 | -12.0003693 | 2024 | 9 | Fog season (May-Oct) | iNaturalist | Citizen-science observation | HUMAN_OBSERVATION | — | 8 |
| 53 | -76.634154 | -12.0003693 | 1964 | 11 | Dry season (Nov-Apr) | F | Herbarium specimen | PRESERVED_SPECIMEN | Lima | — |
| 54 | -76.9530559 | -12.1193961 | 2025 | 10 | Fog season (May-Oct) | iNaturalist | Citizen-science observation | HUMAN_OBSERVATION | — | 6 |
| 55 | -76.930598 | -12.130625 | — | — | Undated | Bioversity | Field/genebank record | OCCURRENCE | — | — |
| 56 | -76.3826257 | -12.1328708 | 1990 | 10 | Fog season (May-Oct) | CUVC | Field/genebank record | OCCURRENCE | En la entrada a San Jos de los Chorrillos, carretera de Cieneguilla hacia Huarochir. | — |
| 57 | -76.9283522 | -12.1351166 | 2025 | 10 | Fog season (May-Oct) | iNaturalist | Citizen-science observation | HUMAN_OBSERVATION | — | 8 |
| 58 | -76.9283522 | -12.1373624 | 2025 | 10 | Fog season (May-Oct) | iNaturalist | Citizen-science observation | HUMAN_OBSERVATION | — | 2 |
| 59 | -76.9238607 | -12.1373624 | 2025 | 7 | Fog season (May-Oct) | iNaturalist | Citizen-science observation | HUMAN_OBSERVATION | — | 3 |
| 60 | -76.9216149 | -12.1373624 | 2022 | 3 | Dry season (Nov-Apr) | iNaturalist | Citizen-science observation | HUMAN_OBSERVATION | — | 180 |
| 61 | -76.930598 | -12.1396082 | 2023 | 5 | Fog season (May-Oct) | iNaturalist | Citizen-science observation | HUMAN_OBSERVATION | — | 198 |
| 62 | -76.602713 | -12.1396082 | 2025 | 5 | Fog season (May-Oct) | iNaturalist | Citizen-science observation | HUMAN_OBSERVATION | — | — |
| 63 | -76.9238607 | -12.1440997 | 2025 | 9 | Fog season (May-Oct) | iNaturalist | Citizen-science observation | HUMAN_OBSERVATION | — | — |
| 64 | -76.2321579 | -12.1688034 | 2023 | 10 | Fog season (May-Oct) | iNaturalist | Citizen-science observation | HUMAN_OBSERVATION | — | 80 |
| 65 | -76.8385207 | -12.1890155 | 2021 | 1 | Dry season (Nov-Apr) | iNaturalist | Citizen-science observation | HUMAN_OBSERVATION | — | 245 |
| 66 | -76.8362749 | -12.1912613 | 2023 | 7 | Fog season (May-Oct) | iNaturalist | Citizen-science observation | HUMAN_OBSERVATION | — | 560 |
| 67 | -76.8115713 | -12.1912613 | 2025 | 10 | Fog season (May-Oct) | iNaturalist | Citizen-science observation | HUMAN_OBSERVATION | — | 20 |
| 68 | -76.8340291 | -12.1935071 | 2023 | 9 | Fog season (May-Oct) | iNaturalist | Citizen-science observation | HUMAN_OBSERVATION | — | 4 |
| 69 | -76.8317834 | -12.1935071 | 2022 | 10 | Fog season (May-Oct) | iNaturalist | Citizen-science observation | HUMAN_OBSERVATION | — | 10 |
| 70 | -76.8295376 | -12.1935071 | 2021 | 3 | Dry season (Nov-Apr) | iNaturalist | Citizen-science observation | HUMAN_OBSERVATION | — | — |
| 71 | -76.8272918 | -12.1935071 | 2021 | 3 | Dry season (Nov-Apr) | iNaturalist | Citizen-science observation | HUMAN_OBSERVATION | — | 30 |
| 72 | -76.8138171 | -12.1935071 | 2025 | 6 | Fog season (May-Oct) | iNaturalist | Citizen-science observation | HUMAN_OBSERVATION | — | 4 |
| 73 | -76.8115713 | -12.1935071 | 2025 | 6 | Fog season (May-Oct) | iNaturalist | Citizen-science observation | HUMAN_OBSERVATION | — | 14 |
| 74 | -76.8093255 | -12.1935071 | 2025 | 2 | Dry season (Nov-Apr) | iNaturalist | Citizen-science observation | HUMAN_OBSERVATION | — | 2422 |
| 75 | -76.8340291 | -12.1957529 | 2024 | 7 | Fog season (May-Oct) | iNaturalist | Citizen-science observation | HUMAN_OBSERVATION | — | — |
| 76 | -76.8228002 | -12.1957529 | 2025 | 4 | Dry season (Nov-Apr) | iNaturalist | Citizen-science observation | HUMAN_OBSERVATION | — | 639 |
| 77 | -76.8183086 | -12.1957529 | 2025 | 11 | Dry season (Nov-Apr) | iNaturalist | Citizen-science observation | HUMAN_OBSERVATION | — | 167 |
| 78 | -76.8070797 | -12.1957529 | 2021 | 8 | Fog season (May-Oct) | iNaturalist | Citizen-science observation | HUMAN_OBSERVATION | — | 1036 |
| 79 | -76.8834365 | -12.1979987 | 2023 | 8 | Fog season (May-Oct) | iNaturalist | Citizen-science observation | HUMAN_OBSERVATION | — | 309 |
| 80 | -76.8138171 | -12.1979987 | 2025 | 8 | Fog season (May-Oct) | iNaturalist | Citizen-science observation | HUMAN_OBSERVATION | — | 357 |
| 81 | -76.8115713 | -12.1979987 | 2025 | 2 | Dry season (Nov-Apr) | iNaturalist | Citizen-science observation | HUMAN_OBSERVATION | — | 570 |
| 82 | -76.8295376 | -12.2002445 | 2021 | 3 | Dry season (Nov-Apr) | iNaturalist | Citizen-science observation | HUMAN_OBSERVATION | — | — |
| 83 | -76.8272918 | -12.2002445 | 2022 | 11 | Dry season (Nov-Apr) | iNaturalist | Citizen-science observation | HUMAN_OBSERVATION | — | 50 |
| 84 | -76.8924196 | -12.2024902 | 2024 | 9 | Fog season (May-Oct) | iNaturalist | Citizen-science observation | HUMAN_OBSERVATION | — | — |
| 85 | -76.8879281 | -12.2024902 | 2023 | 8 | Fog season (May-Oct) | iNaturalist | Citizen-science observation | HUMAN_OBSERVATION | — | 82 |
| 86 | -76.8856823 | -12.2024902 | 2016 | 9 | Fog season (May-Oct) | iNaturalist | Citizen-science observation | HUMAN_OBSERVATION | — | 409 |
| 87 | -76.8272918 | -12.2024902 | 2022 | 11 | Dry season (Nov-Apr) | iNaturalist | Citizen-science observation | HUMAN_OBSERVATION | — | 22 |
| 88 | -76.8856823 | -12.204736 | 2022 | 10 | Fog season (May-Oct) | iNaturalist | Citizen-science observation | HUMAN_OBSERVATION | — | 161 |
| 89 | -76.8811907 | -12.204736 | 2024 | 7 | Fog season (May-Oct) | iNaturalist | Citizen-science observation | HUMAN_OBSERVATION | — | 1832 |
| 90 | -76.8295376 | -12.204736 | 2025 | 6 | Fog season (May-Oct) | iNaturalist | Citizen-science observation | HUMAN_OBSERVATION | — | 3 |
| 91 | -76.825046 | -12.204736 | 2021 | 5 | Fog season (May-Oct) | iNaturalist | Citizen-science observation | HUMAN_OBSERVATION | — | 169 |
| 92 | -76.8138171 | -12.2069818 | 2025 | 10 | Fog season (May-Oct) | iNaturalist | Citizen-science observation | HUMAN_OBSERVATION | — | 60 |
| 93 | -76.8003423 | -12.2092276 | 2022 | 2 | Dry season (Nov-Apr) | iNaturalist | Citizen-science observation | HUMAN_OBSERVATION | — | 6 |
| 94 | -76.750935 | -12.215965 | 2022 | 9 | Fog season (May-Oct) | iNaturalist | Citizen-science observation | HUMAN_OBSERVATION | — | 2120 |
| 95 | -76.6925445 | -12.3282544 | 2021 | 10 | Fog season (May-Oct) | iNaturalist | Citizen-science observation | HUMAN_OBSERVATION | — | 2119 |
| 96 | -76.5308477 | -12.7122842 | 2023 | 7 | Fog season (May-Oct) | iNaturalist | Citizen-science observation | HUMAN_OBSERVATION | — | 637 |
| 97 | -76.5308477 | -12.7167757 | 2025 | 7 | Fog season (May-Oct) | iNaturalist | Citizen-science observation | HUMAN_OBSERVATION | — | 728 |
| 98 | -76.4634741 | -12.7729204 | 2024 | 8 | Fog season (May-Oct) | iNaturalist | Citizen-science observation | HUMAN_OBSERVATION | — | — |
| 99 | -76.4679657 | -12.7751662 | 2023 | 7 | Fog season (May-Oct) | iNaturalist | Citizen-science observation | HUMAN_OBSERVATION | — | 15 |
| 100 | -76.4702115 | -12.777412 | 2025 | 7 | Fog season (May-Oct) | iNaturalist | Citizen-science observation | HUMAN_OBSERVATION | — | — |
| 101 | -76.4724572 | -12.7796578 | 2024 | 6 | Fog season (May-Oct) | iNaturalist | Citizen-science observation | HUMAN_OBSERVATION | — | 4 |

**Table S3.** Full list of the 31 environmental variables initially considered for ensemble species distribution modelling of *Vasconcellea candicans.*

| **Variable** | **Description** | **Unit** | **Symbol** |
| --- | --- | --- | --- |
| Bioclimatic | Annual Mean Temperature | °C | bio01 |
|  | Mean Diurnal Range (Mean of monthly (max temp - min temp)) | °C | bio02 |
|  | Isothermality (bio2/bio7) (×100) | % | bio03 |
|  | Temperature Seasonality (standard deviation ×100) | °C ×100 | bio04 |
|  | Max Temperature of Warmest Month | °C | bio05 |
|  | Min Temperature of Coldest Month | °C | bio06 |
|  | Temperature Annual Range (bio5-bio6) | °C | bio07 |
|  | Mean Temperature of Wettest Quarter | °C | bio08 |
|  | Mean Temperature of Driest Quarter | °C | bio09 |
|  | Mean Temperature of Warmest Quarter | °C | bio10 |
|  | Mean Temperature of Coldest Quarter | °C | bio11 |
|  | Annual Precipitation | mm | bio12 |
|  | Precipitation of Wettest Month | mm | bio13 |
|  | Precipitation of Driest Month | mm | bio14 |
|  | Precipitation Seasonality (Coefficient of Variation) | mm | bio15 |
|  | Precipitation of Wettest Quarter | mm | bio16 |
|  | Precipitation of Driest Quarter | mm | bio17 |
|  | Precipitation of Warmest Quarter | mm | bio18 |
|  | Precipitation of Coldest Quarter | mm | bio19 |
| Topographic | Cardinal orientation of the slope | ° | aspect |
|  | Slope of the terrain | ° | slope |
|  | Elevation above sea level | m a.s.l. | altitude |
| Edaphic at  0.30 m | Apparent density of the fine earth fraction | cg/cm^3^ | bdod |
|  | Cation exchange capacity | cmol kg^-1^ | cec |
|  | Volumetric fraction of coarse fragments | cm^3^ /dm^3^ (vol %) | cfvo |
|  | Total nitrogen | cg/kg | nitrogen |
|  | Clay content | % | clay |
|  | Silt content | % | silt |
|  | Organic carbon | g kg^-1^ | soc |
|  | Soil pH (measured in H₂O suspension) | pH ×10 | phh2o |
|  | Sand content | % | sand |

**Table S4.** Combined high- and moderate-suitability “Area of Interest” (AOI) for *Vasconcellea candicans* under current conditions, by spatial unit.

| **Area / Ecosystem** | **AOI (High+Moderate) km²** | **AOI % of unit** |
| --- | --- | --- |
| Lima's territory | 4923.21 | 14.06% |
| Fog oasis | 798.36 | 34.17% |
| Fragile ecosystem | 340.08 | 57.86% |
| Regional Conservation Areas (RCA) | 29.22 | 20.96% |
| National Protected Areas (NPA) | 44.17 | 2.40% |

**Table S5.** Projected habitat suitability of *Vasconcellea candicans* across all GCM–SSP combinations for 2050 and 2070 in the department of Lima. Values in parentheses indicate percentage change relative to current conditions (1970–2000). Suitability classes are defined as: Low (0.2–0.4), Moderate (0.4–0.6), and High (>0.6). Percentages are calculated relative to the total surface area of the department (35,892.49 km²).

| **Climate scenarios** | | | **High** | | **Moderate** | | **Low** | | **Unsuitable** | |
| --- | --- | --- | --- | --- | --- | --- | --- | --- | --- | --- |
|  |  |  | **km² (Δ%**  **vs.**  **current)** | **% of**  **suitable [%**  **of Lima]** | **km² (Δ%**  **vs.**  **current)** | **% of**  **suitable [%**  **of Lima]** | **km² (Δ%**  **vs.**  **current)** | **% of**  **suitable [%**  **of Lima]** | **km² (Δ%**  **vs.**  **current)** | **% of Lima** |
| Current (1970-2000) | | | 2891.28 | 34.01 [8.26] | 2031.93 | 23.90 [5.80] | 3577.22 | 42.08 [10.22] | 24952.44 | 71.27 |
| 2050s | SSP2–4.5 | ACCESS-CM2 | 3600.28 (+24.52) | 36.97 [10.28] | 2309.17 (+13.64) | 23.72 [6.60] | 3827.64 (+7.00) | 39.31 [10.93] | 23712.22 (−4.97) | 67.73 |
|  |  | IPSL-CM6A-LR | 2818.92 (−2.50) | 33.76 [8.05] | 2010.45 (−1.06) | 24.08 [5.74] | 3519.33 (−1.62) | 42.15 [10.05] | 25100.62 (+0.59) | 71.69 |
|  |  | MIROC6 | 3109.56 (+7.55) | 35.40 [8.88] | 2086.13 (+2.67) | 23.75 [5.96] | 3588.29 (+0.31) | 40.85 [10.25] | 24665.33 (−1.15) | 70.45 |
|  | SSP5–8.5 | ACCESS-CM2 | 3705.43 (+28.16) | 37.23 [10.58] | 2348.86 (+15.60) | 23.60 [6.71] | 3899.15 (+9.00) | 39.17 [11.14] | 23495.87 (−5.84) | 67.11 |
|  |  | IPSL-CM6A-LR | 2848.78 (−1.47) | 33.71 [8.14] | 2052.15 (+1.00) | 24.28 [5.86] | 3549.96 (−0.76) | 42.01 [10.14] | 24998.42 (+0.18) | 71.40 |
|  |  | MIROC6 | 3331.03 (+15.21) | 36.39 [9.51] | 2153.38 (+5.98) | 23.53 [6.15] | 3669.04 (+2.57) | 40.08 [10.48] | 24295.86 (−2.63) | 69.39 |
| 2070s | SSP2–4.5 | ACCESS-CM2 | 3655.92 (+26.45) | 37.49 [10.44] | 2293.11 (+12.85) | 23.52 [6.55] | 3802.46 (+6.30) | 38.99 [10.86] | 23697.82 (−5.03) | 67.69 |
|  |  | IPSL-CM6A-LR | 2773.22 (−4.08) | 33.72 [7.92] | 1978.97 (−2.61) | 24.06 [5.65] | 3471.47 (−2.96) | 42.21 [9.92] | 25225.65 (+1.09) | 72.05 |
|  |  | MIROC6 | 2967.81 (+2.65) | 35.40 [8.48] | 1916.49 (−5.68) | 22.86 [5.47] | 3500.26 (−2.15) | 41.75 [10.00] | 25064.76 (+0.45) | 71.59 |
|  | SSP5–8.5 | ACCESS-CM2 | 4520.45 (+56.35) | 41.69 [12.91] | 2396.34 (+17.93) | 22.10 [6.84] | 3925.49 (+9.74) | 36.21 [11.21] | 22607.03 (−9.40) | 64.57 |
|  |  | IPSL-CM6A-LR | 2880.91 (−0.36) | 34.35 [8.23] | 2010.25 (−1.07) | 23.97 [5.74] | 3494.83 (−2.30) | 41.67 [9.98] | 25063.33 (+0.44) | 71.59 |
|  |  | MIROC6 | 3655.08 (+26.42) | 37.85 [10.44] | 2263.84 (+11.41) | 23.44 [6.47] | 3737.46 (+4.48) | 38.70 [10.68] | 23792.93 (−4.65) | 67.96 |

**Table S6.** Range shift statistics for *Vasconcellea candicans* under future climate change scenarios in comparison to current climate conditions.

| **Scenario** | **Loss (km²)** | **Absent (km²)** | **Stable (km²)** | **Gain (km²)** | **Loss (%)** | **Gain (%)** | **Range change (%)** |
| --- | --- | --- | --- | --- | --- | --- | --- |
| IPSL-CM6A-LR SSP2-4.5 (2050s) | 397.31 | 24704.31 | 8103.76 | 246.01 | 4.67 | 2.89 | -1.78 |
| ACCESS-CM2 SSP5-8.5 (2050s) | 419.58 | 23077.61 | 8081.48 | 1872.71 | 4.94 | 22.03 | +17.09 |
| IPSL-CM6A-LR SSP2-4.5 (2070s) | 495.81 | 24730.80 | 8005.26 | 219.53 | 5.83 | 2.58 | -3.25 |
| ACCESS-CM2 SSP5-8.5 (2070s) | 689.52 | 21919.20 | 7811.55 | 3031.12 | 8.11 | 35.66 | +27.54 |

**Figure S1.** Projected habitat suitability of *Vasconcellea candicans* in the department of Lima across all six GCM–SSP combinations for 2050 and 2070. Upper panels (a–f): 2050s projections under ACCESS-CM2 SSP2-4.5 (a), ACCESS-CM2 SSP5-8.5 (b), IPSL-CM6A-LR SSP2-4.5 (c), IPSL-CM6A-LR SSP5-8.5 (d), MIROC6 SSP2-4.5 (e), and MIROC6 SSP5-8.5 (f). Lower panels (a–f): corresponding projections for the 2070s. Suitability classes Low: 0.2–0.4; Moderate: 0.4–0.6; High: >0.6.

**Table S7**. Landscape metrics of urban expansion in the department of Lima for 1990, 2000, 2010, and 2020. LSI: Landscape Shape Index; FRAC: Fractal Dimension Index; CI: Compactness Index; NP: number of patches; MPS: mean patch size (ha).

| **Year** | **Area (km²)** | **Perimeter (km)** | **LSI** | **FRAC** | **CI** | **NP** | **MPS (ha)** |
| --- | --- | --- | --- | --- | --- | --- | --- |
| 1990 | 426.05 | 1.4 | 63.9706 | 0.9063 | 51.2126 | 1615 | 0.0027 |
| 2000 | 590.66 | 1.7 | 63.2384 | 0.9047 | 51.7966 | 1664 | 0.0036 |
| 2010 | 713.87 | 1.8 | 60.9651 | 0.9059 | 53.7385 | 1355 | 0.0053 |
| 2020 | 842.63 | 1.8 | 55.9552 | 0.9052 | 58.5562 | 1051 | 0.0081 |

**Table S8**. Inter-decadal changes (Δ) in landscape metrics of urban expansion in the department of Lima for the periods 1990–2000, 2000–2010, and 2010–2020, relative to the preceding time step.

| **Period** | **Δ Area (km²)** | **Δ Perimeter (km)** | **Δ LSI** | **Δ FRAC** | **Δ CI** | **Δ NP** | **Δ MPS (ha)** |
| --- | --- | --- | --- | --- | --- | --- | --- |
| **1990–2000** | 164.61 | 0.3 | -0.7322 | -0.0016 | 0.584 | 49 | 0.0009 |
| **2000–2010** | 123.21 | 0.1 | -2.2733 | 0.0012 | 1.9419 | -309 | 0.0017 |
| **2010–2020** | 128.76 | 0 | -5.0099 | -0.0007 | 4.8177 | -304 | 0.0028 |

**Figure S2.** Land use and land cover distribution in the department of Lima for observed years (a) 1990, (b) 2000, (c) 2010, and (d) 2020, and projected scenarios for (e) 2050 and (f) 2070, derived from the Land Change Modeler (LCM). Urban area and fog oasis are shown as primary land cover classes.

**Table S9.** Spatial overlap between suitable habitat and urban expansion.

| **Suitability class** | **Period** | **SDM → SDM (km²)** | **SDM → Urban (km²)** | **Without class → Urban (km²)** | **Habitat loss (%)** |
| --- | --- | --- | --- | --- | --- |
| High | 2020 | 2855.16 | 36.12 | 806.64 | 1.25 |
|  | 2050 | 2791.59 | 99.69 | 1186.06 | 3.45 |
|  | 2070 | 2764.34 | 126.94 | 1436.13 | 4.39 |
| Moderate | 2020 | 2004.90 | 27.03 | 815.73 | 1.33 |
|  | 2050 | 1970.20 | 61.73 | 1224.03 | 3.04 |
|  | 2070 | 1946.16 | 85.77 | 1477.30 | 4.22 |
| Low | 2020 | 3525.39 | 51.83 | 790.94 | 1.45 |
|  | 2050 | 3470.22 | 106.99 | 1178.76 | 2.99 |
|  | 2070 | 3433.12 | 144.10 | 1418.97 | 4.03 |
| Unsuitable | 2020 | 24794.90 | 157.54 | 685.23 | 0.63 |
|  | 2050 | 24549.57 | 402.87 | 882.89 | 1.61 |
|  | 2070 | 24380.39 | 572.05 | 991.02 | 2.29 |

**Table S10.** Habitat suitability distribution (km²) for *Vasconcellea candicans* across key spatial units under current conditions (2020).

| **Area / Ecosystem** | **High (>0.6)** | | **Moderate**  **(0.4-0.6)** | | **Low (0.2-0.4)** | | **Total km^2^** | **Total %** |
| --- | --- | --- | --- | --- | --- | --- | --- | --- |
|  | **km^2^** | **%** | **km^2^** | **%** | **km^2^** | **%** |  |  |
| Fog oasis | 584.43 | 26.12 | 213.93 | 9.56 | 262.28 | 11.72 | 1060.64 | 47.41 |
| Fragile ecosystem | 275.39 | 47.32 | 64.69 | 11.12 | 67.21 | 11.55 | 407.30 | 69.98 |
| Regional Conservation Areas (RCA) | 21.12 | 15.68 | 8.10 | 6.01 | 7.73 | 5.74 | 36.95 | 27.43 |
| National Protected Areas (NPA) | 21.70 | 1.18 | 22.47 | 1.22 | 108.91 | 5.93 | 153.09 | 8.34 |

**Table S11.** Habitat suitability distribution (km²) for *Vasconcellea candicans* across key spatial units under IPSL-CM6A-LR SSP2-4.5 for 2050.

| **Area / Ecosystem** | **High (>0.6)** | | **Moderate**  **(0.4-0.6)** | | **Low (0.2-0.4)** | | **Total km^2^** | **Total %** |
| --- | --- | --- | --- | --- | --- | --- | --- | --- |
|  | **km^2^** | **%** | **km^2^** | **%** | **km^2^** | **%** |  |  |
| Fog oasis | 606.34 | 27.13 | 208.12 | 9.31 | 253.66 | 11.35 | 1068.13 | 47.80 |
| Fragile ecosystem | 278.14 | 47.82 | 63.57 | 10.93 | 66.92 | 11.50 | 408.62 | 70.25 |
| Regional Conservation Areas (RCA) | 22.11 | 16.42 | 7.68 | 5.70 | 9.06 | 6.73 | 38.85 | 28.84 |
| National Protected Areas (NPA) | 22.86 | 1.25 | 18.26 | 0.99 | 85.52 | 4.66 | 126.63 | 6.90 |

**Table S12.** Habitat suitability distribution (km²) for *Vasconcellea candicans* across key spatial units under ACCESS-CM2 SSP5-8.5 for 2050.

| **Area / Ecosystem** | **High (>0.6)** | | **Moderate**  **(0.4-0.6)** | | **Low (0.2-0.4)** | | **Total km^2^** | **Total %** |
| --- | --- | --- | --- | --- | --- | --- | --- | --- |
|  | **km^2^** | **%** | **km^2^** | **%** | **km^2^** | **%** |  |  |
| Fog oasis | 616.57 | 27.64 | 206.16 | 9.24 | 257.46 | 11.54 | 1080.18 | 48.42 |
| Fragile ecosystem | 277.66 | 47.24 | 66.72 | 11.35 | 69.04 | 11.75 | 413.42 | 70.34 |
| Regional Conservation Areas (RCA) | 23.17 | 17.20 | 8.16 | 6.06 | 9.78 | 7.26 | 41.10 | 30.52 |
| National Protected Areas (NPA) | 28.96 | 1.58 | 46.51 | 2.53 | 195.46 | 10.65 | 270.92 | 14.76 |

**Table S13.** Habitat suitability distribution (km²) for *Vasconcellea candicans* across key spatial units under IPSL-CM6A-LR SSP2-4.5 for 2070.

| **Area / Ecosystem** | **High (>0.6)** | | **Moderate**  **(0.4-0.6)** | | **Low (0.2-0.4)** | | **Total km^2^** | **Total %** |
| --- | --- | --- | --- | --- | --- | --- | --- | --- |
|  | **km^2^** | **%** | **km^2^** | **%** | **km^2^** | **%** |  |  |
| Fog oasis | 608.82 | 27.24 | 207.93 | 9.30 | 255.07 | 11.41 | 1071.82 | 47.96 |
| Fragile ecosystem | 278.61 | 47.90 | 63.65 | 10.94 | 66.65 | 11.46 | 408.90 | 70.30 |
| Regional Conservation Areas (RCA) | 21.87 | 16.24 | 7.92 | 5.88 | 9.12 | 6.77 | 38.91 | 28.89 |
| National Protected Areas (NPA) | 22.43 | 1.22 | 16.24 | 0.88 | 66.96 | 3.65 | 105.63 | 5.75 |

**Table S14.** Habitat suitability distribution (km²) for *Vasconcellea candicans* across key spatial units under ACCESS-CM2 SSP5-8.5 for 2070.

| **Area / Ecosystem** | **High (>0.6)** | | **Moderate**  **(0.4-0.6)** | | **Low (0.2-0.4)** | | **Total km^2^** | **Total %** |
| --- | --- | --- | --- | --- | --- | --- | --- | --- |
|  | **km^2^** | **%** | **km^2^** | **%** | **km^2^** | **%** |  |  |
| Fog oasis | 601.29 | 27.00 | 203.05 | 9.12 | 258.02 | 11.59 | 1062.36 | 47.70 |
| Fragile ecosystem | 269.01 | 45.77 | 67.37 | 11.46 | 72.40 | 12.32 | 408.78 | 69.55 |
| Regional Conservation Areas (RCA) | 23.62 | 17.53 | 8.39 | 6.23 | 10.33 | 7.67 | 42.33 | 31.43 |
| National Protected Areas (NPA) | 54.42 | 2.96 | 71.49 | 3.89 | 200.31 | 10.91 | 326.22 | 17.77 |
